# What the AI era doctor should know: a scoping review of proposed artificial intelligence competencies for medical education

**DOI:** 10.1038/s41746-026-02761-9

**Published:** 2026-05-12

**Authors:** Victor M. Hunt, Laurine K. Sprehe, Weston C. de Lomba, Rodrigo R. Gameiro, Naira L. Woite, Carrie G. Wade, Jonathan H. Chen, Steven C. Rougas, Hamish S. F. Fraser, Amy M. Sullivan, Felipe Fregni

**Affiliations:** 1 The Warren Alpert Medical School, Brown University, Providence, RI USA; 2 Brown Center for Biomedical Informatics, Brown University, Providence, RI USA; 3 Massachusetts General Hospital, Harvard Medical School, Boston, MA USA; 4 Heidelberg University Hospital, Heidelberg University, Heidelberg, Germany; 5 Department of Biomedical Informatics, Harvard Medical School, Boston, MA USA; 6 Institute for Medical Engineering and Science, Massachusetts Institute of Technology, Cambridge, MA USA; 7 Department of Internal Medicine, Yale School of Medicine, New Haven, CT USA; 8 Countway Library, Harvard Medical School, Boston, MA USA; 9 Stanford Division of Computational Medicine, Division of Hospital Medicine, Clinical Excellence Research Center, Stanford University, Stanford, CA USA; 10 Beth Israel Deaconess Medical Center, Shapiro Institute Center for Education, Boston, MA USA; 11 Harvard T.H. Chan School of Public Health, Boston, MA USA

**Keywords:** Computational biology and bioinformatics, Health care, Mathematics and computing, Medical research, Scientific community, Social sciences

## Abstract

Artificial intelligence (AI) is rapidly reshaping healthcare and the competencies expected of graduating medical students, yet AI curricula and competency recommendations for undergraduate medical education (UME) remain fragmented. We conducted a PRISMA-ScR scoping review to map and synthesize proposed AI competencies for UME by performing a global search of PubMed, Embase, Web of Science, and ERIC without language restrictions and from database inception through July 28, 2025. Verbatim competency-relevant text was extracted and decomposed into discrete statements and classified using domains, competencies, or learning objectives. Statement frequencies were summarized to characterize recurring areas of emphasis, underrepresented topics, and cross-domain relationships. Of 4071 records identified, 54 studies from 22 countries met inclusion criteria. From 564 eligible statements, we synthesized a taxonomy comprising seven domains (AI ethics; AI law and regulation; AI professionalism in healthcare; clinical applications of AI; critical appraisal of AI output; research and innovation in AI; theory and foundations of AI) spanning 37 competencies and 170 learning objectives. Sources were predominantly editorial/opinion, with recurring emphasis on ethicolegal oversight, critical appraisal of AI outputs, and foundational understanding of AI methods and data. This synthesis provides a structured inventory to inform curriculum planning and future stakeholder-based refinement, prioritization, and evaluation.

## Introduction

Artificial intelligence (AI) is transforming healthcare and necessitates corresponding updates to medical education curricula. AI broadly refers to computational systems capable of performing tasks that typically require human intelligence. AI has enabled novel digital solutions in diagnostics^1–4^, imaging^5,6^, and clinical decision support and patient management^7–9^. Like the stethoscope, X-ray, and other breakthrough technologies, AI is increasingly positioned to become an everyday tool that shapes physicians’ decision-making on behalf of and in conjunction with their patients.

Multinational survey data show that most patients favor AI technologies that improve efficiency and consistency—so long as they do not displace physician oversight^10^. But with the growing accessibility of AI comes associated risk: while many AI-based healthcare initiatives are in the developmental or pilot stages^11^, in the United States nearly half of hospitals^12^ and up to two-thirds of physicians report using some form of AI in their practice^13^. Without greater accountability and understanding, there is a possibility that widespread issues of generalizability, collaboration, and transparency may grow unchecked within the clinical environment^14^. Models that may perform well during development can degrade in real-world settings due to dataset drift across diverse sites, devices, and populations^15–18^. Physicians must be trained to recognize errors, mixed evidence, and overreliance, as well as how to properly integrate AI into professional workflows. Even among regulated AI products, available evidence and disclosure are often insufficient to assess for bias and/or accuracy, underscoring the need for competency in the critical appraisal of resources^19,20^.

Medical education has not kept pace with these rapid advancements. Existing recommendations remain sparse and heterogeneous^21,22^. Several scoping reviews have examined AI within medical education^23–28^, but these syntheses largely describe broad “AI literacy” themes or program-level content rather than systematically extracting and organizing competency- and learning-objective-level outcomes for undergraduate medical education (UME). Few institutions have implemented an AI curriculum; those that have feature broad variation in depth, breadth, and volition^29^. Recognizing that UME curricula are in constant flux—but always near maximum capacity—changes are necessary to ensure AI competency within clinical practice. As AI continues to be incorporated into clinical workflows, physician fluency is increasingly critical. Though the basic science undergirding AI may seem tangential to clinical medicine, physicians must understand AI insofar as they must understand any other tool. They should be expected to use AI appropriately and judiciously; interpret its results and potential sources of error or bias; and explain when, why, and how it was used when communicating with patients and allied health professionals^30^.

It is therefore timely and critical that medical students understand the principles and limitations of AI before entering the clinical workforce as practicing physicians. This necessitates embedding AI competencies within a standardized UME curriculum.

Through constant-comparison coding and prevalence analysis, this scoping review aimed to systematically identify, categorize, and collate the existing literature on AI competencies in UME to support the development of a standardized, competency-based AI framework for medical students. To our knowledge, no prior review has delivered a frequency-weighted hierarchical map spanning AI competencies for medical education. This synthesis intends to provide curriculum committees, licensing bodies, educators, and researchers with a structured inventory of proposed AI competencies to better inform curriculum planning, content prioritization, and future stakeholder-based refinement and validation in response to the evolving AI-based needs of modern medical practice.

## Results

### Selection of sources of evidence

**Fig. 1:**
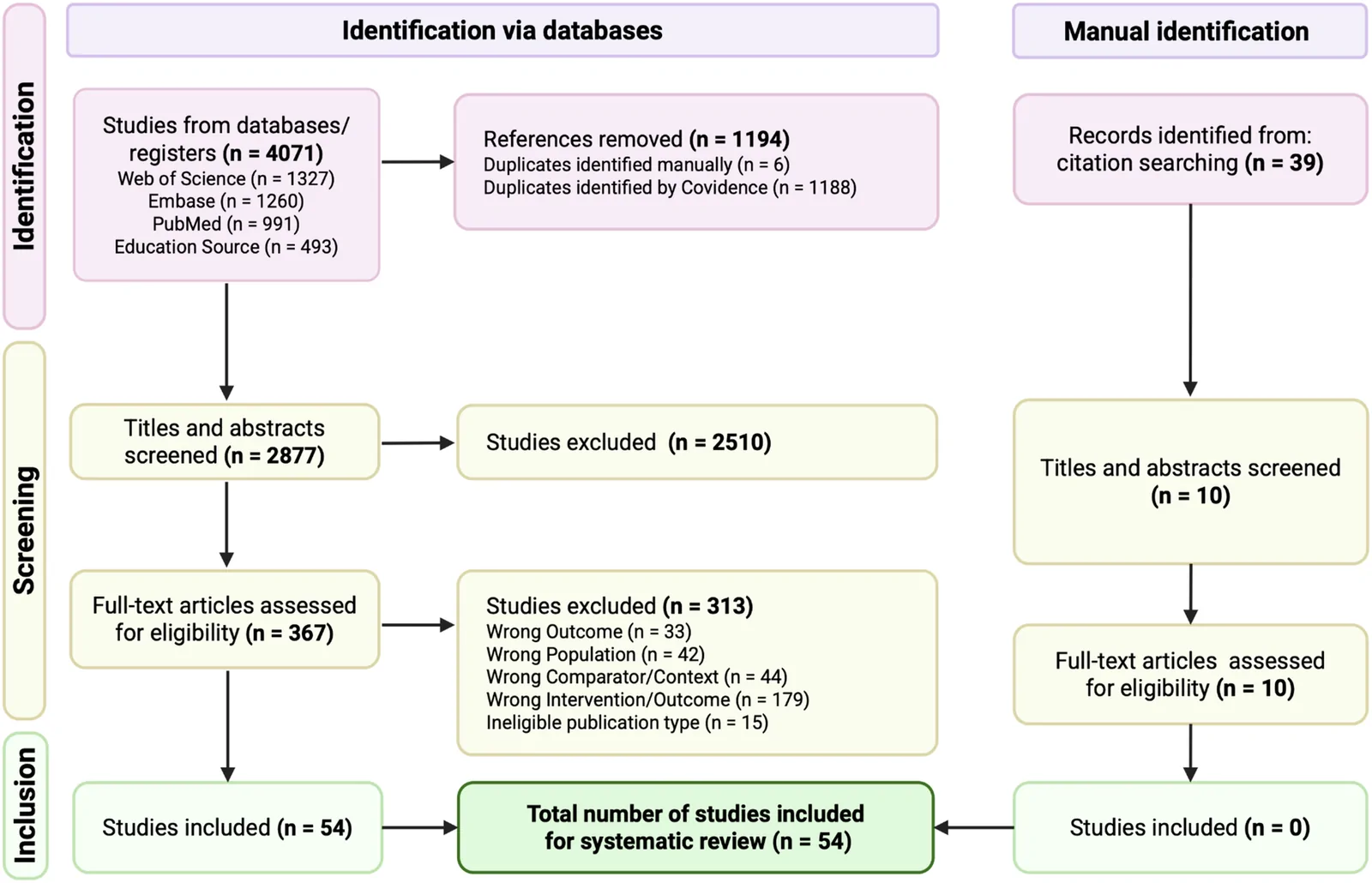
Preferred reporting items for systematic reviews and meta-analyses (PRISMA) flow diagram. Created in BioRender. Publication license: Sprehe, L. (2026). BioRender.com/k2p8w96.

A total of 4071 records were identified. After removal of 1194 duplicates, 2877 titles/abstracts were screened and 367 full texts were assessed for eligibility. Fifty-four studies from 22 countries met inclusion criteria and were included in the scoping review (Fig. 1). Inter-rater reliability was *κ* = 0.32 (87% agreement; *N* = 2877) for title and abstract screening and *κ* = 0.53 (84% agreement; *N* = 367) for full-text eligibility assessment.

### Characteristics of sources of evidence

The 54 included sources were largely recent, with 29 studies (53.7%) published in 2024–2025. Distribution was multinational, with greatest contributions from the United States (16/54, 29.6%) and Germany (6/54, 11.1%). Nearly half of sources were editorial or opinion (24/54, 44.4%), and most described proposed or theoretical competencies (36/54, 66.7%). Aims were commonly framework/competency definition (17/54, 31.5%), with fewer addressing curricular implementation/evaluation (9/54, 16.7%) or curriculum proposal/development (7/54, 13.0%). Few described curricula that were pilot-tested (6/54, 11.1%) or outcome-evaluated (8/54, 14.8%). Where implementation status was specified, most sources recommended mandatory rather than elective curricula (27/54, 50.0%). Stage (preclinical vs. clinical) was typically unspecified (26/54, 48.1%). Aggregate study characteristics are provided in Table 1.

**Table 1:** Characteristics of included studies (*N* = 54)

Characteristic	Overall, *n* (%)
Year of publication	
2019	1 (1.9%)
2020	3 (5.6%)
2021	3 (5.6%)
2022	6 (11.1%)
2023	12 (22.2%)
2024	18 (33.3%)
2025	11 (20.4%)
Country	
United States	16 (29.6%)
Germany	6 (11.1%)
United Kingdom	3 (5.6%)
Canada	3 (5.6%)
China	3 (5.6%)
Singapore	3 (5.6%)
Austria	2 (3.7%)
Japan	2 (3.7%)
Saudi Arabia	2 (3.7%)
Sultanate of Oman	2 (3.7%)
Other (<2 studies)	12 (22.2%)
Citation count	
0–4	18 (33.3%)
5–9	4 (7.4%)
10–49	20 (37.0%)
50–99	9 (16.7%)
100+	3 (5.6%)
0–4	18 (33.3%)
Study aim	
Framework/competency definition	17 (31.5%)
Curriculum implementation/evaluation	9 (16.7%)
Medical education adaptation commentary	7 (13.0%)
Curriculum proposal/development	7 (13.0%)
Needs assessment/perceptions	5 (9.3%)
Ethics/regulatory perspective	3 (5.6%)
Learning objectives specification	1 (1.9%)
Unspecified	5 (9.3%)
Study design	
Editorial/opinion	24 (44.4%)
Cross-sectional survey study	9 (16.7%)
Curriculum proposal	5 (9.3%)
Delphi study (expert consensus)	5 (9.3%)
Quasi-experimental	4 (7.4%)
Mixed methods study (including pilots)	3 (5.6%)
Narrative review	2 (3.7%)
Qualitative (semi-structured interviews)	2 (3.7%)
Curricular stage	
Both	14 (25.9%)
Clinical	7 (13.0%)
Preclinical	7 (13.0%)
Unspecified	26 (48.1%)
Implementation details	
Mandatory	27 (50.0%)
Elective	13 (24.1%)
Unspecified	14 (25.9%)
Implementation gaps/maturity	
Proposed-only / theoretical	36 (66.7%)
Pilot evaluated for outcomes	8 (14.8%)
Pilot-tested in curricula	6 (11.1%)
Unspecified	4 (7.4%)

### Critical appraisal of sources of evidence

Funding and/or support was disclosed in 17/54 sources (31.5%), while 11/54 (20.4%) included disclosures (e.g., consulting or advisory roles, employment, active research grants); detailed study-level disclosures are provided in Supplementary Table 2. Citation counts exhibited right skew with most studies accruing few citations and a small number cited broadly.

### Results of individual sources of evidence

Study-level charted data for each included source of evidence, including full citation and study characteristics relevant to the review question, are presented in Supplementary Table 2.

### Synthesis of results

Verbatim content extracted from the 54 included studies was disaggregated into 633 preliminary competency statements. After statement-level relevance screening by three reviewers (full consensus), 564 statements remained for synthesis. Level classification inter-rater reliability was *κ* = 0.55 (70% agreement).

**Fig. 2:**
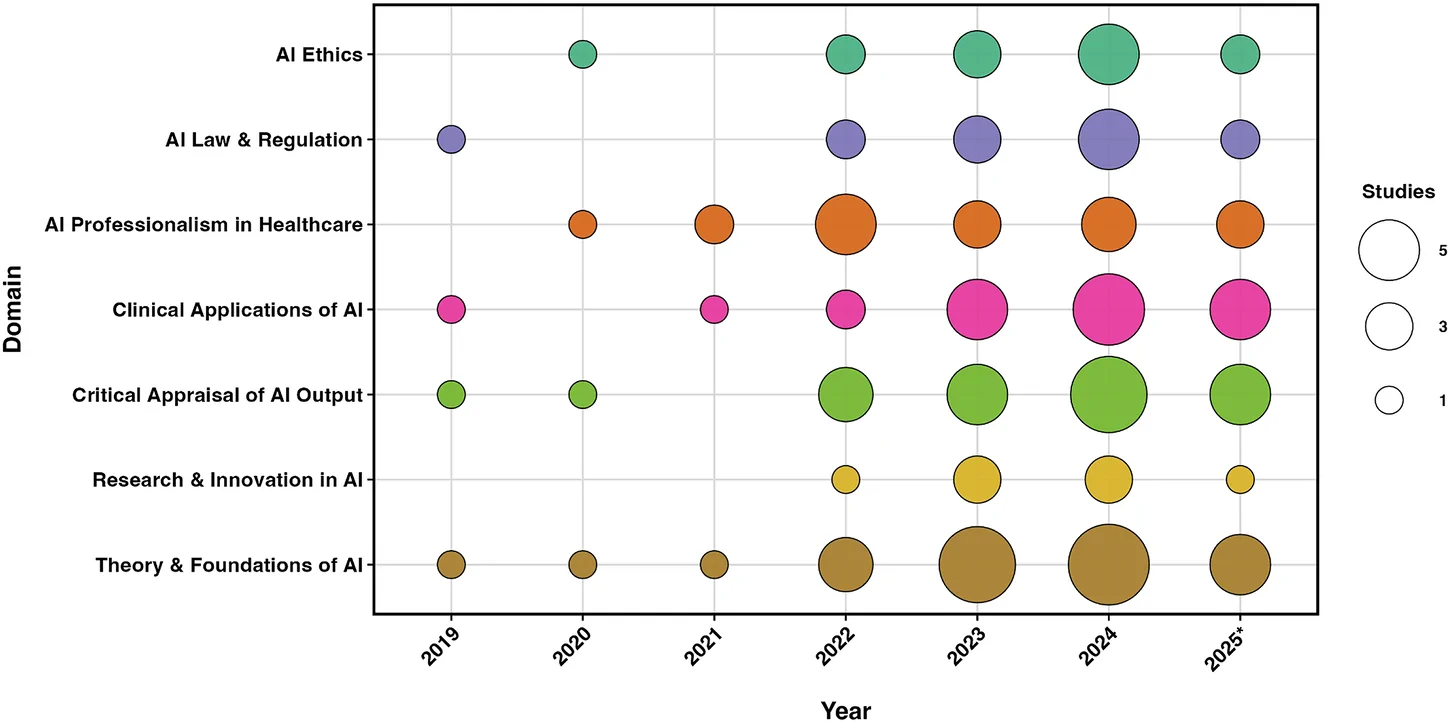
Evidence gap map showing the distribution and volume of published studies across key artificial intelligence (AI) education domains over time. Each bubble represents the number of studies within a specific domain–year combination, with bubble size proportional to study count. Studies that address multiple domains are included in each corresponding domain row. *2025 includes studies published through July 28, 2025.

**Fig. 3:**
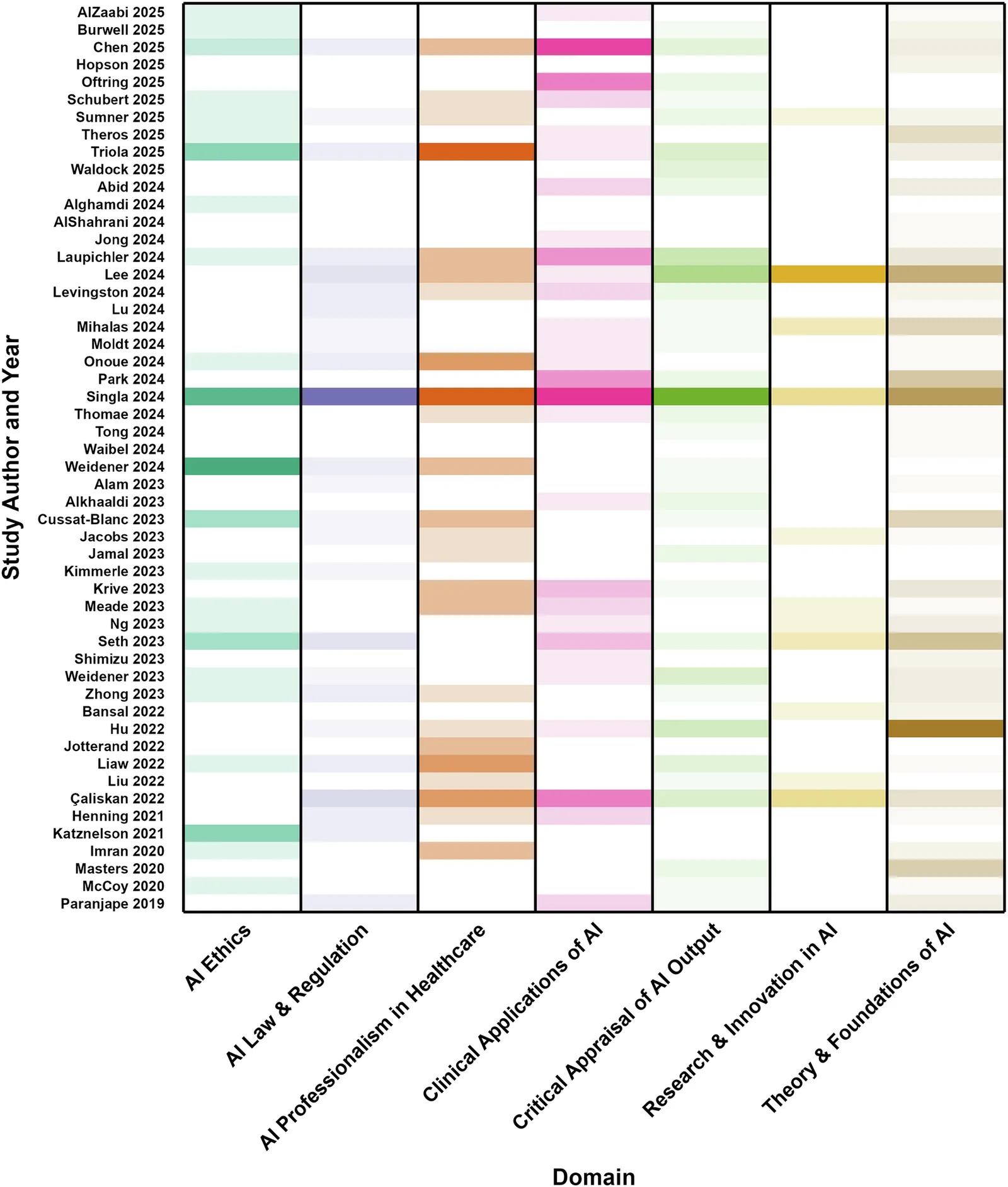
Total extracted competency statements per study by domain. Heat map displaying the number of eligible extracted competency statements (total sum of competencies and learning objectives) within each of the seven domains across 54 included studies. Each row represents a study and each row represents a domain. Color intensity is normalized across domains, with maximal intensity reflecting the highest number of extracted statements observed for that domain across all studies.

Domain-level statements (*n* = 64) were thematically clustered to generate provisional labels, which through reviewer consensus were then consolidated into seven domains. All 564 statements were then assigned to one of these domains with high inter-rater reliability (*κ* = 0.82; 85% agreement). The final taxonomy comprised seven domains—AI Ethics, AI Law and Regulation, AI Professionalism in Healthcare, Clinical Applications of AI, Critical Appraisal of AI Output, Research and Innovation in AI, and Theory and Foundations of AI—which together encompassed 37 competencies represented by 170 learning objectives. Figure 2 depicts domain-level evidence coverage over time using study counts by domain-year; studies addressing multiple domains were counted in each applicable domain. Overall, domain coverage expanded markedly after 2022, with the largest study volumes concentrated in Theory & Foundations of AI, Critical Appraisal of AI Output, and Clinical Applications of AI, whereas Research & Innovation in AI appeared less frequently across years. Distribution of statement density across domains and competencies at the study level is shown in Fig. 3, which demonstrates substantial between-study heterogeneity, with domain hotspots driven by a small number of statement-dense studies—most notably within Clinical Applications and AI Law & Regulation—while Research & Innovation remained sparse across most sources.

Within each domain, competency-level statements (*n* = 161) were clustered to derive consolidated competency labels (Table 2). Learning objective-level statements (*n* = 338) were subsequently grouped within competency clusters (*k* = 37) to derive consolidated learning objective clusters (*k* = 170), which were labeled in an inductive, reviewer-led synthesis. A full table of the synthesized undergraduate medical education AI competency taxonomy including domains and their nested competencies and learning objectives can be found in Supplementary Table 3. In sensitivity analyses excluding editorial/opinion studies, all seven domains and all 37 competencies remained represented. Domain and competency emphasis rankings were highly correlated with the full dataset (domains *ρ* = 0.89; competencies *ρ* = 0.88), with modest rank shifts concentrated in clinically-oriented versus appraisal/professionalism competencies (Supplementary Table 4). Learning objective support decreased modestly (19.4% attrition, 33/170) with losses as follow: AI Ethics 5/13, AI Law and Regulation 4/17, AI Professionalism 3/15, Clinical Applications 5/30, Critical Appraisal 9/33, Research & Innovation 2/13, and Theory and Foundations 5/49.

**Table 2:** Synthesized undergraduate medical education AI competency framework domains and competencies

Domain	Competency
Theory and foundations of AI *N* = 46 studies	1. AI foundations and core concepts (*N* = 20) 2. Data science and health data fundamentals (*N* = 14) 3. Machine learning concepts and paradigms (*N* = 14) 4. Statistical foundations for AI in medicine (*N* = 9) 5. Biases of AI systems (*N* = 8) 6. Deep learning and neural networks (*N* = 7) 7. Model development, training, and optimization (*N* = 7) 8. Data preprocessing and feature engineering (*N* = 6) 9. Natural language processing and generative AI (*N* = 6) 10. Algorithms and model types (*N* = 4) 11. Evaluation, error analysis, and explainability (*N* = 3)
AI ethics *N* = 37 studies	1. Responsibility, transparency, and patient rights (*N* = 14) 2. Bias and equity (*N* = 10) 3. Allocation and deployment of AI (*N* = 5) 4. Data ethics (*N* = 5)
Critical appraisal of AI output *N* = 35 studies	1. Opportunities and limitations of AI systems (*N* = 15) 2. Analysis, data quality, and methodological Fit (*N* = 14) 3. Evidence appraisal and clinical interpretation (*N* = 12) 4. Model performance, validation, and error characteristics (*N* = 9) 5. Implementation, efficiency, and quality assurance (*N* = 8)
Clinical applications of AI *N* = 34 studies	1. AI-augmented workflow optimization (*N* = 12) 2. Clinical use and selection of AI tools (*N* = 12) 3. AI-based clinical decision support (*N* = 11) 4. Diagnosis, imaging, and pathology (*N* = 7) 5. AI in population health and value-based care (*N* = 3)
AI law and regulation *N* = 26 studies	1. Data privacy/confidentiality and protections (*N* = 12) 2. Regulatory frameworks and approval processes (*N* = 11) 3. Data stewardship (*N* = 7) 4. Legal liability and accountability (*N* = 6) 5. Consent and socio-legal obligations (*N* = 5)
AI professionalism in healthcare *N* = 24 studies	1. Digital communication, conduct, and collaboration (*N* = 15) 2. Physician-patient communication and shared decision-making (*N* = 11) 3. Professional identity, roles, and responsibilities (*N* = 7) 4. Reflective use of AI in patient-centered care (*N* = 5)
Research and innovation in AI *N* = 13 studies	1. Research design and methods (*N* = 7) 2. Research ecosystem and impact (*N* = 4) 3. Applications to translational research (*N* = 3)

Seven domains and their constituent competencies derived from qualitative synthesis organized by frequency of thematic occurrence by study. “*N*” indicates the number of studies contributing at least one eligible competency statement mapped to that construct.

**Fig. 4:**
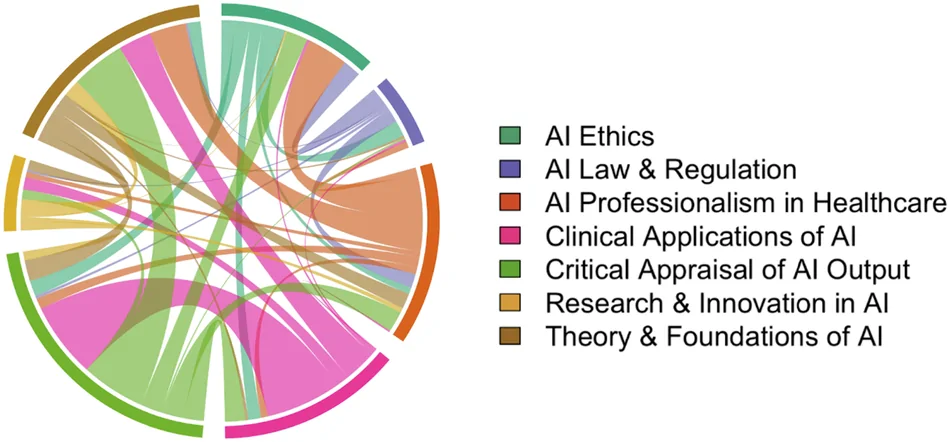
Chord diagram of cross-domain relationships in the synthesized artificial intelligence (AI) competency framework. The diagram visualizes cross-domain linkages mediated by synthesized learning objectives. Outer-ring sectors denote the seven domains, and inner-ring sectors denote competencies nested within their parent domain; only competencies observed to participate in at least one cross-domain linkage are displayed. Chords represent learning objective–mediated connections between competencies across domains, weighted using a study-normalized scheme in which each study contributes a total weight of 1 distributed across its eligible learning objectives, and learning objectives mapped to multiple competencies (limited to one per domain) contribute proportionally to each corresponding cross-domain linkage. Domains are color-coded as follows: AI ethics (teal), AI law and regulation (purple), AI professionalism in healthcare (orange), clinical applications of AI (pink), critical appraisal of AI output (green), research and innovation in AI (yellow), and theory and foundations of AI (brown).

Linkage analysis (Fig. 4) showed the synthesized taxonomy was dominated by two directional pathways among domains: Clinical Applications of AI most strongly referenced competencies within Critical Appraisal of AI Output (weight 5.08), and critical appraisal most strongly invoked competencies within theory and foundations of AI (weight 3.65). Consistent with these patterns, the strongest inbound connectivity was observed for theory and foundations (10.45) and critical appraisal (9.07), indicating that competencies in other domains frequently drew on these areas. The strongest outbound connectivity originated from critical appraisal (8.65) and clinical applications (8.61), suggesting that these domains most often linked outward to others. Full cross-domain linkage values are provided in Supplementary Table 5.

## Discussion

Previous reviews have established that AI is increasingly present in medical education and that trainees will require new competencies to use it safely. Within undergraduate medical education, Lee et al. and Simoni et al. found that most publications describe general content areas rather than detailed competency-based outcomes^23,31^. Cheng et al. and broader scoping work across the medical education continuum (Gordon et al.; Tolentino et al.; Ma et al.; Shaw et al.; Rincón et al.) have articulated high-level domains, mapped curricula and interventions, and emphasized the need for clearer competency frameworks^24–27,32,33^. Other scoping reviews (Preiksaitis and Rose) have focused on generative AI and AI ethics, and policy statements (IFMSA) have begun to outline expected AI-related skills^28,34^.

Building on this foundation, the present review moves from broad themes to specific outcomes by treating competency and learning objective statements as the unit of analysis. Across 54 included studies, we extracted 564 competency-relevant statements and synthesized them into a hierarchical taxonomy comprising 7 domains (AI ethics, AI law and regulation, AI professionalism in healthcare, clinical applications of AI, critical appraisal of AI output, research and innovation in AI, and theory and foundations of AI), spanning 37 competencies and 170 learning objectives. This synthesis directly addresses the review objective by consolidating disparate competency proposals into a structured taxonomy spanning foundational AI concepts, clinical applications, and socioeconomic considerations (ethics, professionalism, and regulations) to support curriculum planning, content prioritization, and future stakeholder-based refinement and validation. Yet the evidence base remains predominantly propositional. In sensitivity analysis excluding editorial/opinion pieces, domain and competency rank-ordering remained highly correlated with the full dataset, suggesting that the overall structure of the taxonomy is not driven solely by opinion sources. A subset of learning objectives lost support when restricted to non-editorial studies and was most pronounced in Critical Appraisal and Ethics-adjacent objectives, consistent with editorials preferentially emphasizing reflective or interpretive themes. Overall, there remains limited reporting on curricular implementation and evaluation of learner outcomes. In this context, heterogeneity in how competencies are defined, organized and operationalized likely contributes to ongoing challenges in establishing a widely adopted, internationally shared framework for undergraduate AI medical education.

Domains and competencies synthesized here may help inform curricular development and future approaches to assessing student AI competencies across institutions. While several competencies with comparatively modest representation included implementation, efficiency, and quality assurance, and the research and innovation constructs (Research design and methods; Research ecosystem and impact), they may still be important to the safe and effective use of AI in clinical settings. This is of particular salience in the present moment given the rapid uptake of general-purpose and domain-specific generative AI tools within the clinical workspace with opaque safety and bias characteristics and in advance of a robust evidence base. Competency in recognizing these levels and limitations of evidence for any AI system in patient care is therefore essential and aligns with Critical Appraisal competencies. Beyond this, the growing use of LLMs by patients and community members elevates the importance of Physician–Patient Communication and Shared Decision-making and Reflective Use of AI in Patient-Centered Care, including counseling on benefits, risks, and appropriate use across patient-, population- and community-level contexts. Linkage analysis of the synthesized taxonomy suggests potential points for curricular integration: clinical-use competencies repeatedly invoke appraisal and foundation concepts, suggesting that schools may draw on these patterns by embedding evidence appraisal and core AI literacy alongside applied clinical AI use cases, rather than teaching these areas in isolation.

In practice, these linkage patterns support an integrated approach to implementation. Applied clinical AI use cases can be taught alongside explicit instruction in critical appraisal and core AI foundations, since these domains are frequently linked in the literature. Medical schools can operationalize this taxonomy by mapping high-frequency competencies onto existing curricular structures (e.g., evidence-based medicine, clinical reasoning, health systems science, professionalism or ethics). Using case-based sessions, students can interpret AI outputs, articulate limitations, and learn to communicate these implications to patients. A longitudinal model with early foundational exposure with reinforcement during clerkships may be more feasible than a standalone course in an otherwise crowded UME curriculum. Because prevalence reflects publication emphasis rather than intrinsic priority, these counts should be used as a starting point for local sequencing and resource allocation rather than a prescriptive ranking. For curriculum committees, licensing bodies, educators, and researchers, this review may inform near-term curriculum development efforts while in tandem underscoring the urgent need for stakeholder refinement and prioritization, implementation studies, and rigorous evaluation of competency attainment and educational outcomes.

Several limitations should be noted inherent to the scoping approach and to the emerging nature of the underlying literature. We did not perform risk-of-bias assessment, consistent with scoping review objectives. Consequently, the synthesis reflects the range of proposed competencies rather than the quality or effectiveness of any educational approach. Accordingly, the resulting taxonomy should be interpreted as a synthesis of published proposals rather than a validated or stakeholder-endorsed competency framework. Included studies varied widely in how they defined and structured competencies, learning outcomes, and curricular components, and many provided limited implementation detail, precluding the ability to draw strong conclusions about optimal implementation across UME or feasibility in specific contexts. The competency statement decomposition and classification required interpretive judgment and although we used multiple-reviewer screening with reviewer consensus, inter-rater reliability was modest at early screening and moderate for level classification, reflecting both ambiguity in source reporting and conceptual overlap across competency areas. Machine translation supported eligibility assessment and extraction for non-English sources, therefore some nuance may have been lost. As most sources posited theoretical curricula and approaches were heterogeneous, prevalence patterns reflect literature emphasis rather than intrinsic priority among competencies.

Taken together, literature most frequently emphasized foundational AI concepts and data, ethicolegal oversight, and critical appraisal of AI outputs and evidence, indicating that these are the most frequently proposed of an AI UME curriculum. This taxonomy was derived from a systematic multi-database search with verbatim statement-level extraction, independent multi-reviewer classification with quantified agreement, and study-normalized prevalence and linkage mapping, allowing identification of recurrent areas of emphasis and comparatively underrepresented topics (e.g., implementation, quality assurance, research, and evidence appraisal methods). While the international community has proposed a variety of recommendations for UME, it is evident that both overlaps and gaps exist. This work underscores the need for collaboration among key stakeholders to refine, prioritize, and validate AI-related competencies across the diverse educational contexts of the AI-era undergraduate medical student. Next steps include multi-stakeholder validation and prioritization (e.g., Delphi processes including educators, students, clinicians, informaticians, patients, and regulators) to define minimum core competencies, refine terminology, and adapt implementation guidance across contexts. Empirical studies are also needed to evaluate instructional models and assessment strategies linked to competency attainment and downstream clinical performance.

## Methods

This review was conducted using Arksey and O’Malley’s framework for scoping reviews^35^ and reported according to the PRISMA extension for scoping reviews (PRISMA-ScR)^36^.

### Protocol registration

The review protocol was registered on the Open Science Framework (OSF; registration t62r5) and is publicly accessible. No deviations from the registered protocol were made.

### Eligibility criteria

Eligibility was defined using the Population–Concept–Context (PCC) framework^37^. We included peer-reviewed literature from any setting that proposed original AI-related competencies (including skills, learning objectives, or curriculum components) explicitly intended for undergraduate medical students; mixed-learner sources were included only when medical-student competencies were reported separately. Sources were required to explicitly reference AI or related technologies (e.g., machine learning). We excluded those sources focused exclusively on postgraduate trainees or other health professions, those addressing digital health informatics without explicit mention of AI content, and any non-original editorials and/or commentaries. Editorials and commentaries were eligible when they proposed original, explicit competency or learning objective statements for medical students. Systematic and scoping reviews were excluded, but their references were screened for eligible primary studies.

### Information sources and search strategy

To identify potentially relevant records, we searched PubMed, Embase, Web of Science, and ERIC from database inception through July 28, 2025 (final search date), recognizing that conceptual work on AI in medicine and medical informatics predates the recent surge in generative AI. The search strategy was developed, translated across databases, and executed by a medical librarian (C.G.W.) through the Harvard Countway Library review service, in consultation with the investigators, and was refined iteratively by the entire review team. Searches combined controlled vocabulary and keywords for artificial intelligence (e.g., “artificial intelligence,” “machine learning,” etc.) with undergraduate medical education and competency-related concepts (e.g., “medical student,” “competency,” “learning objective,” “curriculum”). No date or language limits were applied. For the small amount of studies that did not have an English version available, machine translation was used to support eligibility determination as needed. The electronic database search was supplemented by review article reference screening. The full search strategy is provided in Supplementary Table 1.

### Selection of sources of evidence

Title/abstract and full-text screening were conducted by the review team (V.M.H., L.K.S., W.C.D., R.R.G., N.L.W.) using Covidence (Veritas Health Innovation, Melbourne, Australia). At least two reviewers independently screened titles and abstracts and assessed full texts, and all discrepancies were resolved by consensus with a third reviewer. Inter-rater reliability for each screening stage was assessed using Cohen’s kappa.

### Data charting process

A standardized data charting form was developed in Covidence a priori and piloted on a subset of included studies by at least two reviewers with iterative refinement to improve structure, clarity, and consistency before full extraction. Study-level extraction was performed by L.K.S., including verbatim competency-related text from all available sections (main text, tables, appendices/supplements). Completeness was verified by W.C.D., with discrepancies resolved by consensus among V.M.H., L.K.S, and W.C.D. Machine translation was used to support verbatim extraction as needed. For the small number of non-English full texts included, translation accuracy was verified by a native speaker on the team (German-language full text; L.K.S.). Once extraction was completed, all further data charting and syntheses were managed using shared, version-controlled Google Sheets (Google LLC, Mountain View, CA, USA) with documented iterative updates.

### Data items

Data items extracted from each included study included bibliometrics, author metadata, and study characteristics relevant to educational synthesis, including study aims and design, and curricular stage and implementation details. Furthermore, all verbatim AI-competency–relevant text was extracted from each included source, including content presented in narrative text, tables, figures, appendices, and supplementary materials.

### Critical appraisal of individual sources of evidence

No formal critical appraisal or risk-of-bias assessment was performed as this scoping review aimed to map and synthesize proposed AI competency content rather than evaluate net effects. Funding, disclosures, and citation counts were charted descriptively for context and were not used for study exclusion or to weight the synthesis.

### Synthesis of results

Extracted text was decomposed into discrete competency statements (“statements”), defined as the smallest unit of text that endorsed a distinct AI-related skill, knowledge area, concept, or ability referenced in the source literature. Statements then underwent a statement-level relevance screen for explicit AI content, conducted by multiple independent reviewers with consensus resolution. All statements were assigned by at least two reviewers to provisional “domains”; following a round of reviewer consensus, formal domain labels were then derived. Statement-level classification proceeded deductively utilizing well-established hierarchical competency-framework schemata; taxonomy development for domains, competencies, and learning objectives proceeded inductively through constant-comparison thematic synthesis, guided by educational theory (e.g., Bloom’s taxonomy).

For the final analytic set, two reviewers applied an a priori rubric (Supplementary Note 1) to independently perform level classification that categorize each statement as a domain-, competency-, or learning-objective-level item, with third reviewer adjudication. Disagreements were resolved by adjudication. Domain labels and competency clusters were derived using constant-comparison thematic synthesis with iterative, reviewer-led consolidation with all coding decisions recorded in version-controlled spreadsheets. Quantitative summaries used descriptive prevalence counts and study-normalized frequencies of both statements and proposed synthesis labels to support tabular and figure-based reporting. As a sensitivity analysis, we repeated study-support and prevalence calculations after excluding studies classified as editorial/opinion pieces. We compared domain and competency emphasis between the full and restricted datasets using rank-order stability (Spearman *ρ*) and tabulated rank changes.

### Ethics

Ethics approval was not required, as this study synthesized data from publicly available sources and involved no human participants.

## Supplementary information

Revision Supplementary Material

## Data Availability

The datasets (including full verbatim extraction and statement-level coding spreadsheets) will be made available in the Open Science Framework repository (OSF; registration t62r5).

## Code availability

The underlying code/scripts used to process the extracted data and generate the analyses and visualizations reported in this study will be made available in the Open Science Framework repository (OSF; registration t62r5)^38^.

## References

[1] Fraser, H. et al. Comparison of diagnostic and triage accuracy of Ada Health and WebMD symptom checkers, ChatGPT, and physicians for patients in an emergency department: clinical data analysis study. *JMIR Mhealth Uhealth* **11**, e49995 (2023). 10.2196/49995 PMC10582809 37788063

[2] Liu, H. et al. A deep learning model integrating mammography and clinical factors facilitates the malignancy prediction of BI-RADS 4 microcalcifications in breast cancer screening. *Eur. Radiol.* **31**, 5902–5912 (2021). 10.1007/s00330-020-07659-y 33496829

[3] Gulshan, V. et al. Development and validation of a deep learning algorithm for detection of diabetic retinopathy in retinal fundus photographs. *JAMA* **316**, 2402–2410 (2016). 10.1001/jama.2016.17216 27898976

[4] Abràmoff, M. D., Lavin, P. T., Birch, M., Shah, N. & Folk, J. C. Pivotal trial of an autonomous AI-based diagnostic system for detection of diabetic retinopathy in primary care offices. *npj Digit. Med.* **1**, 39 (2018). 10.1038/s41746-018-0040-6 PMC6550188 31304320

[5] De Fauw, J. et al. Clinically applicable deep learning for diagnosis and referral in retinal disease. *Nat. Med.* **24**, 1342–1350 (2018). 10.1038/s41591-018-0107-6 30104768

[6] McKinney, S. M. et al. International evaluation of an AI system for breast cancer screening. *Nature* **577**, 89–94 (2020). 10.1038/s41586-019-1799-6 31894144

[7] Komorowski, M., Celi, L. A., Badawi, O., Gordon, A. C. & Faisal, A. A. The artificial intelligence clinician learns optimal treatment strategies for sepsis in intensive care. *Nat. Med* **24**, 1716–1720 (2018). 10.1038/s41591-018-0213-5 30349085

[8] Guo, Y. et al. Evaluating ambient artificial intelligence documentation: effects on work efficiency, documentation burden, and patient-centered care. *J. Am. Med. Inf. Assoc.* **33**, 273–282 (2026). 10.1093/jamia/ocaf180 PMC12844569 41100159

[9] Olson, K. D. et al. Use of ambient AI scribes to reduce administrative burden and professional burnout. *JAMA Netw. Open* **8**, e2534976 (2025). 10.1001/jamanetworkopen.2025.34976 PMC12492056 41037268

[10] Busch, F. et al. Multinational attitudes Toward AI in health care and diagnostics among hospital patients. *JAMA Netw. Open* **8**, e2514452 (2025). 10.1001/jamanetworkopen.2025.14452 PMC12152705 40493367

[11] Sahni, N. R. & Carrus, B. Artificial intelligence in U.S. Health Care Delivery. *N. Engl. J. Med.* **389**, 348–358 (2023). 10.1056/NEJMra2204673 37494486

[12] Hwang, Y.M., Ng, M. Y., Pillai, M., Sahai, M. P. & Hernandez-Boussard, T. The landscape of AI implementation in US hospitals. *Nat. Health* **1**, 99–112 (2026).

[13] Angus, D. C. et al. AI, health, and health care today and tomorrow: the JAMA Summit Report on Artificial Intelligence. *JAMA* **334**, 1650–1664 (2025). 10.1001/jama.2025.18490 41082366

[14] Rajpurkar, P. & Lungren, M. P. The current and future state of AI interpretation of medical images. *N. Engl. J. Med.* **388**, 1981–1990 (2023). 10.1056/NEJMra2301725 37224199

[15] Yanase, J. & Triantaphyllou, E. A systematic survey of computer-aided diagnosis in medicine: past and present developments. *Expert Syst. Appl.* **138**, 112821 (2019).

[16] Elhaddad, M. & Hamam, S. AI-driven clinical decision support systems: an ongoing pursuit of potential. *Cureus* **16**, e57728 (2024). 10.7759/cureus.57728 PMC11073764 38711724

[17] Gentile, F. & Malara, N. Artificial intelligence for cancer screening and surveillance. *ESMO Real. World Data Digit. Oncol.* **5**, 100046 (2024). 10.1016/j.esmorw.2024.100046 PMC12836662 41648648

[18] Geaney, A. et al. Translation of tissue-based artificial intelligence into clinical practice: from discovery to adoption. *Oncogene* **42**, 3545–3555 (2023). 10.1038/s41388-023-02857-6 PMC10673711 37875656

[19] Celi, L. A., Dorotic, M., Dubin, J., Nazarian, N. & Salarikia, R. Epistemic humility for physicians and scientists. *Lancet Reg. Health Am.* **53**, 101315 (2026). 10.1016/j.lana.2025.101315 PMC12681921 41362748

[20] Lin, J. C. et al. Benefit-risk reporting for FDA-cleared artificial intelligence-enabled medical devices. *JAMA Health Forum* **6**, e253351 (2025). 10.1001/jamahealthforum.2025.3351 PMC12475944 41004181

[21] Lee, Y.M. et al. Defining medical AI competencies for medical school graduates: outcomes of a Delphi survey and medical student/educator questionnaire of South Korean Medical Schools. *Acad. Med.* **99**, 524–533 (2024). 10.1097/ACM.0000000000005618 38207056

[22] Singla, R. et al. Developing a Canadian artificial intelligence medical curriculum using a Delphi study. *npj Digit. Med.* **7**, 323 (2024). 10.1038/s41746-024-01307-1 PMC11574260 39557985

[23] Lee, J., Wu, A. S., Li, D. & Kulasegaram, K. M. Artificial intelligence in undergraduate medical education: a scoping review. *Acad. Med.* **96**, S62–S70 (2021). 10.1097/ACM.0000000000004291 34348374

[24] Gordon, M. et al. A scoping review of artificial intelligence in medical education: BEME Guide No. 84. *Med. Teach.* **46**, 446–470 (2024). 10.1080/0142159X.2024.2314198 38423127

[25] Tolentino, R., Baradaran, A., Gore, G., Pluye, P. & Abbasgholizadeh-Rahimi, S. Curriculum frameworks and educational programs in AI for medical students, residents, and practicing physicians: scoping review. *JMIR Med. Educ.* **10**, e54793 (2024). 10.2196/54793 PMC11294785 39023999

[26] Shaw, K., Henning, M. A. & Webster, C. S. Artificial intelligence in medical education: a scoping review of the evidence for efficacy and future directions. *Med. Sci. Educ.* **35**, 1803–1816 (2025). 10.1007/s40670-025-02373-0 PMC12228863 40625971

[27] Rincón, E. H. H. et al. Mapping the use of artificial intelligence in medical education: a scoping review. *BMC Med. Educ.* **25**, 526 (2025). 10.1186/s12909-025-07089-8 PMC11993958 40221725

[28] Preiksaitis, C. & Rose, C. Opportunities, challenges, and future directions of generative artificial intelligence in medical education: scoping review. *JMIR Med. Educ.* **9**, e48785 (2023). 10.2196/48785 PMC10625095 37862079

[29] Ng, F. Y. C. et al. Artificial intelligence education: an evidence-based medicine approach for consumers, translators, and developers. *Cell Rep. Med.* **4**, 101230 (2023). 10.1016/j.xcrm.2023.101230 PMC10591047 37852174

[30] McCoy, L. G. et al. What do medical students actually need to know about artificial intelligence? *npj Digit. Med.* **3**, 86 (2020). 10.1038/s41746-020-0294-7 PMC7305136 32577533

[31] Simoni, J. et al. Artificial intelligence in undergraduate medical education: an updated scoping review. *BMC Med. Educ.* **25**, 1609 (2025). 10.1186/s12909-025-08188-2 PMC12625756 41250109

[32] Cheng, D. et al. Framework for AI integration into the undergraduate medical curricula: a scoping review. *BMC Med. Educ.* 10.1186/s12909-026-08620-1 (2026). PMC13011678 41578265

[33] Ma, Y. et al. Promoting AI competencies for medical students: a scoping review on frameworks, programs, and tools. Preprint at 10.48550/arXiv.2407.18939 (2024).

[34] International Federation of Medical Students’ Associations. The Use of Artificial Intelligence in Medical Practice and Research. https://ifmsa.org/wp-content/uploads/2024/11/The-Use-of-Artificial-Intelligence-in-Medical-Practice-and-Research.pdf (accessed 3 Feb 2026), (IFMSA, 2024).

[35] Arksey, H. & O’Malley, L. Scoping studies: towards a methodological framework. *Int. J. Soc. Res. Methodol.* **8**, 19–32 (2005).

[36] Tricco, A. C. et al. PRISMA extension for scoping reviews (PRISMA-ScR): checklist and explanation. *Ann. Intern. Med.* **169**, 467–473 (2018). 10.7326/M18-0850 30178033

[37] Peters, M. D. J. et al. Updated methodological guidance for the conduct of scoping reviews. *JBI Evid. Synth.* **18**, 2119–2126 (2020). 10.11124/JBIES-20-00167 33038124

[38] Gameiro, R. & DeLomba, W. What should the AI era doctor know? A review of proposed artificial intelligence competencies for medical education. https://osf.io/vja2c/overview (2025). 10.1038/s41746-026-02761-9 PMC13615973 42120544

