# Supplementary material for: What the AI era doctor should know: a scoping review of proposed artificial intelligence competencies for medical education
Source: NPJ Digit Med. 2026 May 12;9:732. doi: 10.1038/s41746-026-02761-9 (PMC13615973; doi:10.1038/s41746-026-02761-9)
Supplement: Supplementary file 1 [file 41746_2026_2761_MOESM1_ESM.pdf]

Supplementary Material — What Should the AI Era Doctor Know? A Scoping Review of Proposed Artificial Intelligence Competencies for Medical Education

Table Of Contents

Supplementary Table 1. Search Strategy 2

Supplementary Table 2. Characteristics of Included Sources 5

Supplementary Note 1. Operational Definitions, Coding Guidance & Analytic Details 11

Supplementary Table 3. Synthesized Competency Taxonomy and Study Support...................... 14

Supplementary Table 4. Sensitivity Analysis – Rank-Order Stability 26

Supplementary Table 5. Domain Cross-Connectivity Matrix 28

Supplementary References. Included Sources of Evidence (*N* = 54) 29

Supplementary Table 1. Search Strategy
Search date: 7/28/2025

DATABASE (PLATFORM)	SEARCH	RESULTS
**PUBMED (NLM)**	("Competency-Based Education"[Mesh] OR "Curriculum"[majr:noexp] OR competency[tiab] OR competencies[tiab] OR proficient[tiab] OR proficiency[tiab] OR proficiencies[tiab] OR framework*[tiab] OR AI-concept[tiab] OR AI-concepts[tiab] OR AI-principle*[tiab] OR AI-strateg*[tiab] OR standard[tiab] OR standards[tiab] OR literate[tiab] OR literacy[tiab] OR benchmark*[tiab] OR bench-mark*[tiab] OR learning-goal*[tiab] OR learning-target*[tiab] OR learning-outcome*[tiab] OR educat*-outcome*[tiab] OR educat*-goal*[tiab] OR AI-curricul*[tiab] OR delphi-study[tiab]) AND ("Artificial Intelligence"[Majr] OR AI[tiab] OR artificial*-intelligen*[tiab] OR GenAI[tiab] OR machine-learning[tiab] OR algorithm*-bias[tiab] OR ((algorithm*[tiab] OR chatbot*[tiab] OR LLM[tiab]) AND (literacy[tiab] OR literate[tiab] OR literacies[tiab] OR ethic*[tiab]))) AND ("Education, Medical"[Mesh] OR "Schools, Medical"[Mesh] OR "students, medical"[mesh] OR "Faculty, medical"[majr] OR medical-educat*[tiab] OR medical-school*[tiab] OR medical-college*[tiab] OR "school of medicine"[tiab] OR "college of medicine"[tiab] OR medical-student*[tiab] OR medic*-trainee* OR trainee*-doctor*[tiab] OR AAMC[tiab] OR medical-curricul*[tiab] OR clinical*-curricul*[tiab] OR medic*-curricul*[tiab] OR undergraduate-medical*[tiab])	990
**EMBASE.COM (ELSEVIER)**	1. 'education'/mj OR 'Competency based education'/exp/mj OR 'curriculum'/mj OR 'goal-directed learning'/exp/mj OR (competency OR competencies OR proficiency OR proficiencies OR framework* OR frame-work* OR standard$ OR literate OR literacy OR literacies OR benchmark* OR bench-mark* OR ((artificial-intelligen* OR AI) NEAR/3 (concept* OR principle* OR strateg* OR curricul* OR educat*)) OR ((learning OR educat* OR curricul*) NEAR/1 (goal$ OR outcome* OR target*)) OR delphi-study):ti,ab,kw<br><br>2. 'Medical Education'/exp OR 'Medical School'/mj OR 'Medical student'/mj OR (((medical* OR medicine) NEAR/2 (college$ or school$ OR student$ OR curricul* OR educat* OR undergrad* OR trainee*)) OR AAMC OR trainee*-doctor*):ti,ab,kw<br><br>3. 'Artificial intelligence'/exp/mj OR 'large language model'/mj/exp OR (AI OR artificial*-intelligen* OR GenAI OR machine-learning OR ((algorithm* OR chatbot OR LLM OR chat-bot) NEAR/3 (litera* OR ethic* OR bias* OR principle* OR standard*))):ti,ab,kw<br><br>4. #1 AND #2 AND #3 AND [embase]/lim	1260
**WEB OF SCIENCE – SCI-EXP, CPCI-S, ESCI, SSHI**	2. TI=(competency OR competencies OR proficiency OR proficiencies OR framework* OR frame-work* OR standard$ OR literate OR literacy OR literacies OR benchmark* OR bench-mark* OR ((artificial-intelligen* OR AI) NEAR/3 (concept* OR principle* OR strateg* OR curricul* OR educat*)) OR ((learning OR educat* OR curricul*) NEAR/1 (goal$ OR outcome* OR target*)) OR delphi-study) OR AB=(competency OR competencies OR proficiency OR proficiencies OR framework* OR frame-work* OR standard$ OR literate OR literacy OR literacies OR benchmark* OR bench-mark* OR ((artificial-	1327

	intelligen* OR AI) NEAR/3 (concept* OR principle* OR strateg* OR curricul* OR educat*)) OR ((learning OR educat* OR curricul*) NEAR/1 (goal$ OR outcome* OR target*)) OR delphi-study) OR AK=(competency OR competencies OR proficiency OR proficiencies OR framework* OR frame-work* OR standard$ OR literate OR literacy OR literacies OR benchmark* OR bench-mark* OR ((artificial-intelligen* OR AI) NEAR/3 (concept* OR principle* OR strateg* OR curricul* OR educat*)) OR ((learning OR educat* OR curricul*) NEAR/1 (goal$ OR outcome* OR target*)) OR delphi-study)<br><br>2. TI=(((medical* OR medicine) NEAR/2 (college$ or school$ OR student$ OR curricul* OR educat* OR undergrad* OR trainee*)) OR AAMC OR trainee*-doctor*) OR AB=(((medical* OR medicine) NEAR/2 (college$ or school$ OR student$ OR curricul* OR educat* OR undergrad* OR trainee*)) OR AAMC OR trainee*-doctor*) OR AK=(((medical* OR medicine) NEAR/2 (college$ or school$ OR student$ OR curricul* OR educat* OR undergrad* OR trainee*)) OR AAMC OR trainee*-doctor*)<br><br>3. TI=(AI OR artificial*-intelligen* OR GenAI OR machine-learning OR ((algorithm* OR chatbot OR LLM OR chat-bot) NEAR/3 (litera* OR ethic* OR bias* OR principle* OR standard*))) OR AB=(AI OR artificial*-intelligen* OR GenAI OR machine-learning OR ((algorithm* OR chatbot OR LLM OR chat-bot) NEAR/3 (litera* OR ethic* OR bias* OR principle* OR standard*))) OR AK=(AI OR artificial*-intelligen* OR GenAI OR machine-learning OR ((algorithm* OR chatbot OR LLM OR chat-bot) NEAR/3 (litera* OR ethic* OR bias* OR principle* OR standard*)))<br><br>4. #1 AND #2 AND #3	
**EDUCATION SOURCE (EBSCO)**	1. DE ("Outcome-based education" OR "Competency tests (Education)" OR "Criterion referenced tests" OR "Outcome assessment (Education)" OR "Educational outcomes" OR "Medical school curriculum" OR "Medical education standards") TI (competency OR competencies OR proficiency OR proficiencies OR framework* OR frame-work* OR standard OR standards OR literate OR literacy OR literacies OR benchmark* OR bench-mark* OR ((artificial-intelligen* OR AI) N3 (concept* OR principle* OR strateg* OR curricul* OR educat*)) OR ((learning OR educat* OR curricul*) N1 (goal OR goals OR outcome* OR target*)) OR delphi-study) AB (competency OR competencies OR proficiency OR proficiencies OR framework* OR frame-work* OR standard OR standards OR literate OR literacy OR literacies OR benchmark* OR bench-mark* OR ((artificial-intelligen* OR AI) N3 (concept* OR principle* OR strateg* OR curricul* OR educat*)) OR ((learning OR educat* OR curricul*) N1 (goal OR goals OR outcome* OR target*)) OR delphi-study) OR KW (competency OR competencies OR proficiency OR proficiencies OR framework* OR frame-work* OR standard OR standards OR literate OR literacy OR literacies OR benchmark* OR bench-mark* OR ((artificial-intelligen* OR AI) N3 (concept* OR principle* OR strateg* OR curricul* OR educat*)) OR ((learning OR educat* OR curricul*) N1 (goal OR goals OR outcome* OR target*)) OR delphi-study)<br><br>2. DE ("Medical school curriculum" OR "Medical education" OR "Anesthesiology education" OR "Clinical clerkship" OR "Clinical medical education" OR "Family medicine education" OR "Medical preceptorship" OR	493

	"Medical schools" OR "Obstetrics education" OR "Medical education policy" OR "Medical education standards" OR "Medical ethics education" OR "Medical school administration" OR "Medical school faculty" OR "Medical school graduates" OR "Medical school curriculum" OR "Medical school accreditation" OR "Medical students" OR "Women medical students") OR TI (((medical* OR medicine) N2 (college* or school* OR student* OR curricul* OR educat* OR undergrad* OR trainee*)) OR AAMC OR trainee*-doctor*) OR AB (((medical* OR medicine) N2 (college* or school* OR student* OR curricul* OR educat* OR undergrad* OR trainee*)) OR AAMC OR trainee*-doctor*) OR KW (((medical* OR medicine) N2 (college* or school* OR student* OR curricul* OR educat* OR undergrad* OR trainee*)) OR AAMC OR trainee*-doctor*)<br><br>3. DE ("Algorithmic bias" OR "Artificial intelligence" OR "Machine learning") OR TI(AI OR artificial*-intelligen* OR GenAI OR machine-learning OR ((algorithm* OR chatbot OR LLM OR chat-bot) N3 (litera* OR ethic* OR bias* OR principle* OR standard*)) OR AB(AI OR artificial*-intelligen* OR GenAI OR machine-learning OR ((algorithm* OR chatbot OR LLM OR chat-bot) N3 (litera* OR ethic* OR bias* OR principle* OR standard*)) OR KW (AI OR artificial*-intelligen* OR GenAI OR machine-learning OR ((algorithm* OR chatbot OR LLM OR chat-bot) N3 (litera* OR ethic* OR bias* OR principle* OR standard*))<br><br>4. S1 AND S2 AND S3	
**TOTAL**		4070
**AFTER DEDUPLICATION**		

Supplementary Table 2. Characteristics of Included Sources

Author Year	Funding	COI	Country	Citations*	Study Aim	Study Design	Curricular Stage	Implementation	Gaps and Maturity
Abid 2024	Not Reported	Grant / research support	United States	17	Curriculum implementation / evaluation	Mixed methods	Clinical	Elective	Pilot evaluated for outcomes
Alam 2023	None	None	Brunei	75	Unspecified / Unclear	Editorial / opinion	Both	Mandatory	Pilot-tested in curricula
Alghamdi 2024	Institutional support program	Not Reported	Saudi Arabia	31	Needs assessment / perceptions	Cross-sectional / survey	Both	Unspecified / NA	Proposed-only / theoretical
Alkhaaldi 2023	Not Reported	Not Reported	United Arab Emirates		Unspecified / Unclear	Cross-sectional / survey	Unspecified / NA	Unspecified / NA	Unspecified
AlShahrani 2024	Institutional support program	None	Saudi Arabia	10	Needs assessment / perceptions	Cross-sectional / survey	Both	Unspecified / NA	Unspecified
AlZaabi 2025	None	None	Sultanate of Oman	1	Needs assessment / perceptions	Cross-sectional / survey	Preclinical	Mandatory	Pilot-tested in curricula
Bansal 2022	None	None	India	6	Medical education adaptation commentary	Editorial / opinion	Both	Mandatory	Proposed-only / theoretical
Burwell 2025	None	None	United States	0	Curriculum proposal / development	Editorial / opinion	Both	Mandatory	Proposed-only / theoretical
Çaliskan 2022	Not Reported	Not Reported	Turkey	89	Framework / competency definition	Delphi	Unspecified / NA	Unspecified / NA	Proposed-only / theoretical

Chen 2025	Govt grant	None	China	0	Framework / competency definition	Delphi	Unspecified / NA	Mandatory	Proposed-only / theoretical
Cussat-Blanc 2023	Govt grant	None	France	11	Medical education adaptation commentary	Editorial / opinion	Unspecified / NA	Elective	Proposed-only / theoretical
Henning 2021	None	None	Germany	15	Medical education adaptation commentary	Editorial / opinion	Unspecified / NA	Unspecified / NA	Proposed-only / theoretical
Hopson 2025	None	None	United States	1	Curriculum implementation / evaluation	Quasi-experimental	Both	Elective	Pilot evaluated for outcomes
Hu 2022	None	None	Canada	60	Curriculum implementation / evaluation	Quasi-experimental	Both	Unspecified / NA	Pilot evaluated for outcomes
Huang 2025	None	None	United States	3	Curriculum proposal / development	Editorial / opinion	Clinical	Elective	Pilot-tested in curricula
Imran 2020	None	None	Pakistan	74	Unspecified / Unclear	Editorial / opinion	Unspecified / NA	Mandatory	Proposed-only / theoretical
Jacobs 2023	Not Reported	None	United States	14	Unspecified / Unclear	Editorial / opinion	Unspecified / NA	Unspecified / NA	Proposed-only / theoretical
Jamal 2023	None	None	Saudi Arabia	54	UUnspecified / Unclear	Editorial / opinion	Unspecified / NA	Mandatory	Proposed-only / theoretical
Jong 2024	None	None	Singapore	6	Framework / competency definition	Editorial / opinion	Unspecified / NA	Mandatory	Proposed-only / theoretical
Jotterand 2022	None	None	United States	28	Medical education adaptation commentary	Editorial / opinion	Unspecified / NA	Mandatory	Proposed-only / theoretical

Katznelson 2021	Grant support	Not Reported	United States	134	Ethics and regulatory perspective	Editorial / opinion	Both	Unspecified / NA	Proposed-only / theoretical
Kimmerle 2023	None	None	Germany	40	Needs assessment / perceptions	Cross-sectional / survey	Unspecified / NA	Mandatory	Proposed-only / theoretical
Krive 2023	None	None	United States	68	Curriculum proposal / development	Quasi-experimental	Both	Elective	Pilot evaluated for outcomes
Laupichler 2024	Not Reported	None	Germany		Framework / competency definition	Narrative review	Unspecified / NA	Unspecified / NA	Unspecified
Lee 2024	Govt grant	None	South Korea	22	Framework / competency definition	Delphi	Unspecified / NA	Unspecified / NA	Proposed-only / theoretical
Levingston 2024	None	Grant / research support	United States	4	Curriculum proposal / development	Editorial / opinion	Preclinical	Elective	Pilot-tested in curricula
Liaw 2022	Not Reported	Industry gift; Fellowship sponsorship; Employment/board role	United States	58	Framework / competency definition	Editorial / opinion	Both	Mandatory	Proposed-only / theoretical
Liu 2022	None	None	United States	58	Needs assessment / perceptions	Mixed methods	Unspecified / NA	Mandatory	Proposed-only / theoretical
Lu 2024	None	None	China	26	Ethics and regulatory perspective	Narrative review	Unspecified / NA	Unspecified / NA	Proposed-only / theoretical

Masters 2020	None	None	Sultanate of Oman	24	Framework / competency definition	Editorial / opinion	Preclinical	Mandatory	Proposed-only / theoretical
McCoy 2020	None	None	United States	196	Framework / competency definition	Editorial / opinion	Both	Mandatory	Proposed-only / theoretical
Meade 2023	None	None	United States	2	Curriculum implementation / evaluation	Curriculum proposal	Clinical	Elective	Pilot evaluated for outcomes
Mihalas 2024	None	None	Romania	2	Curriculum proposal / development	Curriculum proposal	Preclinical	Elective	Pilot-tested in curricula
Moldt 2024	None	None	Germany	21	Framework / competency definition	Qualitative / semi-structured interviews	Unspecified / NA	Mandatory	Proposed-only / theoretical
Ng 2023	Not Reported	Patent / IP	Singapore	78	Framework / competency definition	Narrative review	Unspecified / NA	Mandatory	Proposed-only / theoretical
Oftring 2025	OA / publishing fund	Founder / shareholder	Germany	1	Curriculum implementation / evaluation	Cross-sectional / survey	Clinical	Elective	Pilot evaluated for outcomes
Onoue 2024	Govt grant	None	Japan	4	Learning objectives specification	Delphi	Preclinical	Mandatory	Proposed-only / theoretical
Paranjape 2019	None	None	Netherlands	589	Framework / competency definition	Editorial / opinion	Both	Mandatory	Proposed-only / theoretical
Park 2024	OA/publishing fund	None	United States	15	Curriculum implementation / evaluation	Quasi-experimental	Preclinical	Elective	Pilot evaluated for outcomes

Saroha 2025	None	None	United Kingdom	4	Curriculum proposal / development	Editorial / opinion	Unspecified / NA	Mandatory	Proposed-only / theoretical
Schubert 2025	None	Advisory / board roles; Grant / research support	United Kingdom	27	Framework / competency definition	Editorial / opinion	Unspecified / NA	Mandatory	Proposed-only / theoretical
Seth 2023	None	Employment; Consulting / advisory	Canada	33	Framework / competency definition	Editorial / opinion	Unspecified / NA	Mandatory	Proposed-only / theoretical
Shimizu 2023	None	None	Japan	48	Medical education adaptation commentary	Cross-sectional / survey	Unspecified / NA	Mandatory	Proposed-only / theoretical
Singla 2024	None	Founder / shareholder; Consulting / advisory	Canada	9	Framework / competency definition	Delphi	Preclinical	Mandatory	Proposed-only / theoretical
Sumner 2025	None	Editorial role	New Zealand	1	Framework / competency definition	Cross-sectional / survey	Unspecified / NA	Mandatory	Proposed-only / theoretical
Theros 2025	None	None	United States	3	Curriculum proposal / development	Curriculum proposal	Unspecified / NA	Elective	Proposed-only / theoretical
Thomae 2024	None	Grant / research support; Advisory / board roles; Honoraria	Switzerland	25	Curriculum implementation / evaluation	Mixed methods	Both	Elective	Pilot evaluated for outcomes
Tong 2024	Govt grant	None	China	14	Medical education adaptation commentary	Editorial / opinion	Unspecified / NA	Unspecified / NA	Proposed-only / theoretical

Triola 2025	None	Grant / research support	United States	19	Medical education adaptation commentary	Editorial / opinion	Both	Mandatory	Proposed-only / theoretical
Waibel 2024	OA / publishing fund	None	Germany	4	Curriculum implementation / evaluation	Curriculum proposal	Clinical	Elective	Pilot-tested in curricula
Waldock 2025	University funding	None	United Kingdom	4	Curriculum implementation / evaluation	Cross-sectional / survey	Clinical	Unspecified / NA	Unspecified
Weidener 2023	None	None	Austria	42	Framework / competency definition	Qualitative / semi-structured interviews	Unspecified / NA	Mandatory	Proposed-only / theoretical
Weidener 2024	None	None	Austria	2	Ethics and regulatory perspective	Editorial / opinion	Clinical	Mandatory	Proposed-only / theoretical
Zhong 2023	None	None	Singapore	6	Framework / competency definition	Editorial / opinion	Unspecified / NA	Unspecified / NA	Proposed-only / theoretical

* Citation counts were taken by cross-referencing the source with Google Scholar metrics and are up-to-date as of completion of Covidence data extraction (10/23/2025).

Supplementary Note 1. Operational Definitions, Coding Guidance & Analytic Details

Classification scheme (domain, competency, learning objective) for competency statements:

- **Domain:**
    - Definition: Broad conceptual category that organizes related abilities. It represents a high-level thematic bucket rather than a specific measurable skill.
    - Criteria:
        - Mostly short noun phrases without verb/action e.g. "Data Literacy" or "Responsible ethics".
        - Very broad in scope where many competencies can reasonably be under it.
        - Not directly "assessable" as written.
        - More of a topic area than a specific action/behavior of a learner.
    - Representative examples: *"data ethics" (id 3), or "ethics of AI" (id 87), "clinical application of AI" (id 338), "AI literacy" (id 448)*
- **Competency**
    - Definition: Integrated and assessable ability combining knowledge, skills, and attitudes. Expresses what a learner should be able to do in practice at a meaningful level of performance. Stronger behavioral component e.g. "demonstrates", "expresses", "shows" etc.
    - Criteria:
        - Single sentence, sometimes containing an action verb describing what a learner can actually do in practice.
        - Integrated ability that requires knowledge + skill + some form of judgment.
        - Can be reasonably assessed through performance e.g. OSCE, project, portfolio, etc..
        - Still too broad to be achieved in a single teaching session / may span multiple teaching activities.
    - Representative examples: *"Model development, training, and evaluation" (id 96), "Defines the basic concepts and terminology of AI" (id 204), "Evaluate the findings of medical data analysis" (id 293)*
- **† [Sub-Competency - level may or may not be used]:**
    - Definition: Granular, behaviorally specific component of a competency. Describes a narrower capability that contributes to achieving a broader competency.
    - Criteria:
        - Like competency is still action-oriented (verb), but may be narrower and more behaviorally-specific than a competency.
        - Represents one "component step" of a broader competency.
        - Can be assessed with targeted tasks or checklists.

        - Could reasonably be taught over 1-2 sessions but still feels more than a single fact or skill.
    - Representative examples: *"Collect and synthesize relevant information from patients and other sources for use in AI analysis." (id 72)*
- **Learning Objective:**
    - Definition: Discrete, instructional target that specifies what a learner should know or do after a teaching activity. Very granular / teachable, and not sufficient on its own to represent full competency. Seems very much like a piece of knowledge that can be assessed on a rubric.
    - Criteria:
        - Wordage is highly-specific and instructionally-oriented, often containing actions and starting with "define," "describe," "list," "apply," etc.
        - Narrow enough to be fully addressed in a single teaching session.
        - Can be directly measured in a simple way e.g., quiz item, brief exercise.
        - Focused on a single concept, skill, or behavior rather than an integrated capability.
    - Representative example: *"Expresses the importance of data collection, analysis, evaluation and safety for the development of AI applications in healthcare." (id 201) "Knows about clinical workflows and associated data collection along with its management, storage, and transmission." (id 389)*

† The rubric included an optional "sub-competency" level intended for use only when a reviewer felt finer-grained structure was necessary to resolve ambiguity within perceived competency or learning objective clusters. During synthesis, this level was not used because any interim four-level hierarchies immediately produced unstable, inconsistently granular groupings. Thus, early on the structure was definitively collapsed to three levels to improve stability across domains; it is retained here for transparency.

Synthesis Definitions of Domains, Competencies, and Learning Objectives

Domains were synthesized as the highest-level thematic groupings used to organize related competencies and learning objectives across the extracted literature; this was informed by hierarchical educational-taxonomy concepts (e.g., Bloom's taxonomy) without adopting any single framework verbatim. Competency labels were synthesized as organizing construct headings that reflect the phrasing of the source literature, whereas action-oriented, learner-demonstrable expectations were operationally synthesized at the learning objective level.

Synthesized Domain Definitions:

- **AI Ethics:** A competency statement which espouses the learning of ethical principles governing the development, deployment, and clinical use of AI, including fairness, transparency, accountability, and protection of patient rights and welfare.
- **AI Law & Regulation:** A competency statement endorsing education of the legal and regulatory rules that constrain AI in healthcare, including privacy/confidentiality, consent, data governance, liability, and regulatory pathways for approval, monitoring, and compliance.
- **AI Professionalism in Healthcare:** A competency statement emphasizing the development of professional roles, behaviors, and communication practices when AI is used in care, including documentation, collaboration, digital literacy, and maintaining patient-centered relationships and trust.
- **Clinical Applications of AI:** A competency statement endorsing education of practical selection / use of AI tools in clinical workflows and decision-making, including appropriate indications, integration into care processes, and understanding where AI can add value (or not) across common clinical tasks.
- **Critical Appraisal of AI Output:** A competency statement endorsing abilities related to evaluation of AI tools and outputs for validity and clinical appropriateness, including evidence appraisal, performance/validation concepts, error modes, data quality/fit, and implementation quality assurance.
- **Research & Innovation in AI:** A competency statement endorsing engagement with AI research and innovation as a student and eventually at a clinician level, including the interpretation and contribution to studies, broad knowledge of translational pathways, and the role of AI within research ecosystems and impact.
- **Theory & Foundations of AI:** A competency statement endorsing the teaching of core conceptual and technical foundations that may eventually be sufficient for informed clinical use and evaluation, including basic ML concepts, data/feature fundamentals, model types, training/validation ideas, and key limitations (without requiring deep computer science expertise).

Analytic details: Individual sources contributed competency-relevant content to collectively inform the synthesis. In constant-comparison synthesis, each new statement is continually compared against previously coded statements to refine labels, merge or split categories, and stabilize clusters over iterative rounds. Depending on the analytic objective, prevalence was summarized at the statement level (e.g., statement-density distributions), the study level (e.g., counts of studies addressing domains/competencies), or via learning-objective–mediated cross-domain linkages (e.g., relationships among competencies), with multi-mapped items permitted to contribute to each applicable category or linkage.

Supplementary Table 3. Synthesized Competency Taxonomy and Study Support

Domains are ordered by the number of unique studies contributing at least one eligible competency object; competencies and learning objectives are ranked within their parent domain/competency using the same study-support metric.

Domain	Competency	Learning Objective
**Theory & Foundations of AI (46)**	AI Foundations and Core Concepts (20)	Explain core artificial intelligence concepts and terminology, including definitions of AI, levels and approaches of AI systems, and distinctions among weak AI, machine learning, and deep learning. (5)
		Describe the historical evolution of artificial intelligence and machine learning, including key developments and methodological milestones. (2)
		Analyze key differences and relationships between artificial intelligence and machine learning. (1)
		Apply basic technological skills to use AI-related software tools and clinical interfaces. (1)
		Describe fundamental concepts in robotics, including system architecture, effectors, and haptic technologies. (1)
		Explain common digital health terminology and describe the structure of the local digital health ecosystem. (1)
	Data Science and Health Data Fundamentals (14)	Explain foundational data science concepts relevant to health care, including data types, big data characteristics, patient-generated health data, and core principles of data aggregation, analysis, and personalization for decision-making. (5)
		Describe the role and importance of data science in healthcare, including its connections to evidence-based medicine, real-world evidence, medication safety, predictive analytics, and artificial intelligence. (3)
		Explain the characteristics, structures, and quality considerations of health data, including structured and unstructured data, missing data, emerging data types, clinical variables, data annotation, and standard medical terminologies. (3)
		Apply data science skills to work with medical datasets, including data importation, curation, quality assessment, governance, exploratory analysis, visualization, and interpretation of trends relevant to patient outcomes. (2)
		Describe the role of health records and diverse data sources in the generation, storage, management, and evolution of health data within clinical workflows. (2)
		Analyze and compare major types of databases and data schemas used in health records systems. (1)

		Analyze healthcare data using advanced visualization techniques, including correlation heatmaps, box plots, and regression analyses. (1)
		Analyze healthcare datasets using exploratory data analysis techniques, including identification of outliers, class balance, and feature data types. (1)
		Explain foundational medical informatics concepts relevant to the development of AI applications in healthcare. (1)
		Explain leading data integration practices required to deploy analytic solutions in healthcare environments. (1)
		Explain principles of health data curation and data quality management. (1)
	Machine Learning Concepts and Paradigms (14)	Evaluate the strengths, limitations, and appropriate healthcare applications of different machine learning approaches. (8)
		Explain foundational concepts and principles of machine learning, including major learning paradigms (supervised, unsupervised, and reinforcement learning), common algorithmic properties (e.g., classification vs. regression, online vs. offline learning), and the theoretical basis of widely used machine learning methods. (1)
		Explain how artificial intelligence systems are trained, including the role of data, learning algorithms, and optimization processes. (1)
		Explain the concept of model selection in machine learning and its role in developing effective predictive models. (1)
	Statistical Foundations for AI in Medicine (9)	Analyze the implications of univariate and multivariate logistic regression models for identifying and interpreting clinical risk factors. (5)
		Apply knowledge of sensitivity and specificity to determine the appropriate use or avoidance of AI-supported diagnostic applications based on clinical requirements. (2)
		Explain and interpret common statistical performance metrics used in medical AI, including confusion matrices, accuracy, sensitivity, specificity, predictive values, F1 score, likelihood ratios, relative risk, and odds ratios. (1)
		Explain foundational statistical and probabilistic concepts relevant to artificial intelligence in medicine, including basic statistical principles, descriptive and inferential statistics, and the role of probability in AI and machine learning systems. (1)
		Explain how model parameters and goodness-of-fit statistics are used to interpret and evaluate statistical and machine learning models in medicine. (1)
		Explain the role of core mathematical concepts, such as linear algebra and calculus, in supporting an elementary understanding of artificial intelligence. (1)

	Biases of AI Systems (8)	Apply data preprocessing and feature engineering techniques within the broader machine learning workflow, including model selection and evaluation. (5)
		Analyze how hyperparameter optimization, model selection, and the "black box" nature of AI systems contribute to model behavior, uncertainty, and potential bias. (1)
		Apply data literacy and data analytics skills to initiate, conduct, and evaluate processes such as input bandwidth reduction in AI systems. (1)
	Deep Learning and Neural Networks (7)	Explain the foundational concepts of deep learning and neural networks, including network structures, neuron and weight representations, learning mechanisms, loss functions, gradient descent, backpropagation, and the evolution of modern deep learning architectures. (5)
		Apply core techniques to develop neural networks, including preparing data, constructing convolutional layers, and training and testing convolutional neural networks in clinical or applied contexts. (1)
		Explain how deep learning techniques are applied to perception tasks, including visual systems and speech recognition. (1)
		Explain the high-level mechanisms by which convolutional neural networks perform classification tasks. (1)
	Model Development, Training, and Optimization (7)	Apply structured approaches to develop and evaluate machine learning models, including problem definition, data collection, model construction, execution using user-friendly tools, and performance evaluation with appropriate metrics. (3)
		Explain the processes involved in developing and training artificial intelligence and machine learning models, including algorithm development, training workflows, application, and evaluation. (3)
		Analyze model design and optimization decisions, including tradeoffs between model complexity and simplicity, hyperparameter tuning, and selection of appropriate loss functions. (1)
	Data Preprocessing and Feature Engineering (6)	Apply data preprocessing and feature engineering techniques within the broader machine learning workflow, including model selection and evaluation. (5)
		Apply data preprocessing and feature engineering techniques—including data labeling, normalization, handling imbalanced data, and extracting predictive features—to improve dataset quality for machine learning. (3)
		Describe core data preprocessing and feature engineering concepts, including feature spaces, data types, data cleaning, normalization, class balancing, feature selection, feature importance, data augmentation, and data transformation. (3)
		Analyze high-dimensional data using techniques such as dimensionality reduction (e.g., principal component analysis) and clustering. (1)

		Apply data preprocessing and feature engineering as part of an end-to-end machine learning workflow, including problem definition, model selection, and validation. (1)
	Natural Language Processing and Generative AI (6)	Explain the foundational concepts and principles of natural language processing and text-generating artificial intelligence, including how large language models are structured, trained, and used to generate language. (3)
		Explain the foundational concepts and principles of natural language processing and text-generating artificial intelligence, including how large language models are structured, trained, and used to generate language. (3) (2)
	Algorithms and Model Types (4)	Describe and differentiate major categories of machine learning algorithms and models, including regression methods, decision trees, ensemble methods, instance-based models, and support vector machines, along with their general benefits, limitations, and use cases.
		Explain the high-level mechanisms by which common models—including linear and logistic regression, decision trees, and random forests—operate to perform prediction and classification tasks.
	Evaluation, Error Analysis, and Explainability (3)	Explain foundational concepts related to the evaluation and explainability of medical artificial intelligence systems, including principles of medical analytics AI, explainable AI, and common evaluation and interpretation techniques such as SHAP and hyperparameter tuning. (2)
		Analyze model performance by executing and interpreting error analyses in machine learning and deep learning systems. (1)
		Evaluate and select appropriate artificial intelligence algorithms for specific problems based on their strengths, limitations, and performance considerations. (1)
**AI Ethics (37)**	Responsibility, Transparency, and Patient Rights (14)	Explain the ethical foundations of responsibility, transparency, and patient rights in the use of artificial intelligence in healthcare, including the importance of privacy, accountability, professional roles, trust, and alignment with core bioethical principles such as beneficence, non-maleficence, autonomy, and justice. (3)
		Analyze ethical considerations related to the deployment of large language models, including situations in which their use may be inappropriate, unsafe, or harmful. (1)
		Apply ethical principles when using artificial intelligence technologies in clinical or educational contexts. (1)
	Bias and Equity (10)	Analyze ethical challenges arising from the use of information science and technology in medicine, including social disparities related to the digital divide. (1)
		Analyze how model pretraining and fine-tuning processes encode bias and propose potential strategies to mitigate bias in AI systems. (1)

		Apply knowledge of AI bias by identifying real-world examples of biased systems and assessing their impact on the effectiveness of AI applications. (1)
		Critically evaluate the ethical responsibilities of clinicians in identifying, managing, and responding to bias in medical AI systems, including determining when AI outputs should be questioned, overridden, or continuously monitored to promote equity and justice. (1)
		Explain the concepts of bias, equity, and fairness in medical artificial intelligence, including common sources of bias, risks to equitable patient care, and reliability concerns arising from data and system limitations. (1)
		Recognize that biases can be introduced at multiple stages of AI system development and deployment. (1)
	Allocation and Deployment of AI (5)	Apply ethical principles to identify appropriate indications for artificial intelligence tools and integrate their outputs into clinical decision-making in ways that promote effectiveness, value, equity, fairness, and justice. (1)
		Explain how the allocation and deployment of artificial intelligence in medicine affect equitable access to care, including ethical considerations related to the use of generative AI tools not specifically designed or trained for healthcare applications. (1)
	Data Ethics (5)	Evaluate artificial intelligence tools by assessing their limitations, potential risks, and associated ethical considerations related to health data use. (1)
		Explain foundational data ethics principles relevant to artificial intelligence in medicine, including data confidentiality, fairness, equity, diversity, inclusion, bias, and ethical debates surrounding the fair use of health and clinical data in AI and generative AI systems. (1)
**Critical Appraisal of AI Output (35)**	Opportunities and Limitations of AI Systems (15)	Demonstrate critical, evidence-based appraisal skills to evaluate the quality of health information sources and digital health interventions. (2)
		Evaluate the opportunities, limitations, benefits, and risks of AI-driven and digital health technologies across patient-, population-, and community-level healthcare contexts. (1)
		Analyze and compare human-centered clinical practices with AI-supported approaches, including diagnostic reasoning and clinical history-taking. (1)
		Demonstrate competencies in assessing the opportunities and limitations of text-generating AI systems. (1)
		Evaluate the strengths, limitations, and appropriate applications of different AI model types, including deep learning approaches, across healthcare contexts. (1)

		Understand diverse sources of health data and their respective benefits and limitations, including administrative, billing and claims, population health, public health, and omics data. (1)
	Analysis, Data Quality and Methodological Fit (14)	Critically evaluate the integrity, reliability, limitations, and clinical applicability of AI-related evidence, including traditional machine learning, generative AI, and robotic technologies. (3)
		Assess whether the data used to train AI systems are representative, clinically relevant, and appropriate for the intended clinical context and use case. (2)
		Evaluate the clinical suitability and applicability of medical AI solutions and devices when deployed in real-world clinical settings. (1)
		Explain the importance of data collection, analysis, evaluation, and safety in the development of AI applications in healthcare. (1)
		Explain the limitations of current AI and machine learning models, including their inability to extrapolate beyond the datasets on which they were trained. (1)
		Explain the rationale and appropriate use of traditional statistical, machine learning, and neural network methodologies for health data analysis. (1)
		Maintain vigilance in identifying and filtering out unreliable or misleading information sources. (1)
		Teach students how to identify and evaluate the credibility of online information sources. (1)
		Train random forest and logistic regression classifiers and analyze their performance using appropriate evaluation metrics. (1)
		Understand the full data analysis pipeline from data acquisition through modeling and evaluation. (1)
	Evidence Appraisal and Clinical Interpretation (12)	Assess the clinical significance, risks, and patient-safety implications of AI outputs, including false-positive and false-negative results and barriers to safe clinical use. (3)
		Critically evaluate the accuracy, limitations, and clinical appropriateness of AI-generated outputs, and clearly communicate their implications and constraints in practice. (3)
		Analyze and interpret AI model inputs and outputs to inform clinical and healthcare decision-making, including appropriate documentation of results for patient care. (2)
		Differentiate between the functions, features, and outputs of AI-related tools and applications, and analyze the data they generate in healthcare contexts. (2)
		Interpret machine learning models used in healthcare applications. (2)

		Recognize when large language model outputs do not align with the broader clinical context and appropriately reject unsafe or misleading outputs. (1)
	Model Performance, Validation, and Error Characteristics (9)	Analyze and interpret the performance of medical AI models using appropriate accuracy metrics, validation techniques, and visualizations to assess reliability and clinical relevance. (4)
		Analyze the limitations of AI models in healthcare, including issues of generalizability, bias, reproducibility, and interpretability, and their implications for clinical deployment. (2)
		Analyze how training data characteristics, modeling approaches, and update cycles influence the outputs, stability, and performance of generative AI models in clinical use. (1)
		Anticipate, recognize, and respond to potential adverse effects of AI-based tools in clinical practice, including unintended consequences. (1)
		Understand how AI-based tools are developed and evaluated across the model lifecycle. (1)
	Implementation, Efficiency, and Quality Assurance (8)	Analyze existing AI applications in healthcare to identify limitations, areas for improvement, and opportunities to enhance system performance. (2)
		Anticipate how patient interactions and experiences may change as AI technologies are implemented in healthcare settings. (1)
		Describe the broader ramifications of technology-mediated patient care. (1)
		Evaluate the economic implications of AI adoption in healthcare, including implementation costs, maintenance requirements, and potential cost savings or revenue generation. (1)
		Evaluate the impact of AI on the quality, efficiency, and effectiveness of healthcare delivery, including domain-specific applications and patient care outcomes. (1)
		Incorporate patient perspectives into the development and refinement of AI systems by integrating patient feedback to improve usability and care experiences. (1)
**Clinical Applications of AI (34)**	AI-Augmented Workflow Optimization (12)	Describe the range of clinical and operational domains in which AI is currently applied to augment healthcare delivery and outcomes. (4)
		Describe the development, validation, and clinical integration processes required to implement AI tools in healthcare settings. (2)
		Apply principles of user-centered design to improve the usability and adoption of AI tools in healthcare. (1)
		Describe clinical scenarios in which AI solutions can augment and improve clinical processes. (1)

		Explain the role of health data integration in enabling effective AI-supported healthcare workflows. (1)
		Identify and differentiate applications of natural language processing in healthcare, including clinical documentation, patient communication, and disease surveillance. (1)
		Identify techniques that facilitate the effective implementation of AI in healthcare workflows. (1)
		Organize clinical or operational workflows in alignment with AI-supported processes. (1)
	Clinical Use and Selection of AI Tools for Healthcare Problem-Solving (12)	Explain the range of artificial intelligence and information technologies used in healthcare, including their context-dependent applicability and potential clinical and administrative uses. (4)
		Apply appropriate artificial intelligence tools in healthcare by selecting, using, and interpreting AI-based information in combination with professional knowledge to support patient care. (3)
		Explain which types of clinical problems can and cannot be addressed by artificial intelligence and how specific AI applications are matched to healthcare problems. (3)
		Analyze how artificial intelligence models are assessed and validated for specific clinical use cases. (1)
		Evaluate which artificial intelligence systems are appropriate for use in healthcare by appraising the supporting evidence and effectiveness in specific contexts. (1)
		Explain the end-to-end process of clinical problem identification, data preparation, model selection, implementation, validation, and deployment of AI solutions. (1)
	AI-Based Clinical Decision Support (11)	Explain the fundamental concepts of clinical decision support systems, including their reliance on data and machine learning methods and their use in healthcare delivery. (3)
		Apply artificial intelligence–based tools and model outputs within clinical workflows to support and inform evidence-based clinical decision-making. (2)
		Analyze the role of healthcare providers in evaluating the appropriateness of clinical decision support systems, including assessment of data quality, bias, and contextual clinical factors. (1)
		Apply the master adaptive learner framework to emerging uses of generative AI, including clinical reasoning support and patient-facing applications. (1)
		Explain decision support as a meta-skill that enables the orchestration of interdisciplinary contributors, including both human clinicians and AI systems. (1)

	Diagnosis, Imaging, and Pathology (7)	Analyze and interpret outputs from AI-based imaging models to assess their diagnostic implications. (1)
		Describe how neural networks are used to predict mutation status or tumor types. (1)
		Describe how wearable technologies generate clinical data and support measurement of vital signs and personalized prediction of laboratory values. (1)
		Describe the implications of univariate and multivariate linear regression models within a specified clinical application. (1)
		Explain how AI chatbots collect, structure, and summarize clinical history information. (1)
		Explain how artificial intelligence supports image-based and histopathological diagnosis, including detection of disease patterns and prediction of clinically relevant features. (1)
		Explain major applications of deep learning in healthcare, including image analysis, natural language processing, and time-series data analysis. (1)
		Explore the future potential and practical implications of artificial intelligence in pathology. (1)
	AI in Population Health and Value-Based Care (3)	Analyze and appraise real-world use cases of applied artificial intelligence in value-based care currently implemented by healthcare systems. (1)
		Apply methods to collect and synthesize relevant patient-level and population-level data from multiple sources for use in artificial intelligence analyses. (1)
		Evaluate community health needs and propose artificial intelligence–enabled solutions to support population health improvement and value-based care. (1)
**AI Law & Regulation (26)**	Data Privacy/Confidentiality and Protections (12)	Analyze the privacy and data security risks associated with submitting sensitive health information to publicly accessible generative AI systems. (1)
		Apply legal, ethical, and professional principles to the appropriate use of patient health data, considering data quality, provenance, sovereignty, and policies required for secure and confidential healthcare. (1)
		Define cybersecurity and describe its relevance to the protection of healthcare data. (1)
		Explain the importance of data privacy and confidentiality in the use of artificial intelligence in healthcare, including how health data may be incorporated into large language models and the implications for data exposure through generative AI outputs. (1)
	Regulatory Frameworks and Approval Processes (11)	Apply relevant legal and regulatory requirements when using artificial intelligence technologies in healthcare settings. (1)

		Describe regulatory approval processes applicable to artificial intelligence technologies used in healthcare. (1)
		Explain the legal and regulatory frameworks governing the use of artificial intelligence in healthcare, including data protection requirements, privacy risks, best practices, and relevant regional and international laws, guidelines, and conventions. (1)
	Data Stewardship (7)	Analyze how regulatory requirements influence data-sharing practices in healthcare. (1)
		Apply principles of medical data management and storage, including electronic medical records, in compliance with relevant regulations, laws, ethical standards, and requirements for protecting personal health information. (1)
		Explain foundational concepts of data governance and data stewardship in the context of artificial intelligence, including key governance frameworks, health data provenance, and their importance for responsible and lawful use of medical data. (1)
		Explain key regulatory issues governing data sharing among healthcare institutions, academic organizations, and private entities. (1)
		Explain technical standards and protocols used for medical data storage and exchange between healthcare information systems. (1)
	Legal Liability and Accountability (6)	Explain legal issues related to the use of medical data in artificial intelligence systems. (1)
		Explain legal liability and accountability issues associated with the use of artificial intelligence in healthcare, including responsibilities arising from shared decision-making, potential AI-related treatment errors, and the roles of clinicians, healthcare institutions, and AI developers. (1)
		Identify key legal issues related to copyright in the development and use of artificial intelligence systems. (1)
	Consent and Socio-legal Obligations (5)	Apply legal and ethical obligations to ensure that patients are adequately informed and provide free and informed consent when machine learning–based predictions, recommendations, or decision-support tools are used in clinical care. (1)
		Explain the legal and ethical requirements of informed consent in the context of artificial intelligence in medicine, including the need for physicians to clearly describe AI-supported treatments, associated risks, and limitations to patients. (1)
**AI Professionalism in Healthcare (24)**	Digital Communication, Conduct and Collaboration (15)	Analyze professional knowledge and skills with appropriate accuracy, including recognition of potential sources of error, bias, or clinical inappropriateness when using digital or AI-supported information. (1)

		Apply digital and artificial intelligence–enabled communication tools to access, evaluate, share, and create knowledge, and to effectively communicate AI-related information with healthcare professionals, patients, and lay audiences. (1)
		Apply principles of digital professionalism by using electronic tools and platforms appropriately for self-directed and collaborative learning, while ensuring professional conduct and confidentiality in all digital communications. (1)
		Explain appropriate etiquette and professional considerations when using generative AI in healthcare and patient communication. (1)
		Explain the importance of collaboration and shared responsibility in the effective and responsible management of artificial intelligence applications in healthcare. (1)
		Explain the professional roles and collaborative responsibilities of medical practitioners when applying information science and technology in partnership with relevant specialists. (1)
	Physician-Patient Communication and Shared Decision-making (11)	Apply patient-centered communication strategies to explain artificial intelligence applications, predictions, and results in healthcare, including what AI tools do, why they are used, how outputs should be interpreted, and how to address patient concerns or misconceptions. (6)
		Apply shared decision-making approaches that incorporate AI-informed evidence, including the meaningful communication of probabilities, in ways that preserve or enhance the clinician–patient relationship. (2)
		Create patient-friendly educational materials to communicate AI-related knowledge and research relevant to their care. (1)
		Explain the importance of shared decision-making in AI-enabled healthcare, including the physician's role and the relevance of AI explainability and transparency in supporting patient understanding and trust. (1)
	Professional Identity, Roles, and Responsibilities (7)	Analyze how artificial intelligence reshapes the physician's professional identity, including which aspects of clinical work may be automated and which responsibilities must remain under human judgment. (2)
		Apply professional guidelines, codes of conduct, and ethical standards to clearly articulate and enact the physician's role in implementing machine learning technologies within routine clinical practice. (2)
		Explain the distinct and complementary roles of physicians, engineers, and allied healthcare professionals in the development, deployment, and use of artificial intelligence in healthcare. (1)
	Reflective Use of AI in Patient-Centered Care (5)	Apply empathetic, patient-centered communication strategies when discussing the use of artificial intelligence in patient care, including the reflective use of generative AI

		tools to enhance understanding, manage emotionally charged conversations, and support patient trust, autonomy, and shared decision-making. (2)
		Explain the importance of empathy and the human dimensions of healthcare in the context of increasing use of artificial intelligence, including the broader social significance of information science and technology in medicine. (2)
**Research & Innovation in AI (13)**	Research Design and Methods (7)	Collect, extract, and manage relevant healthcare data from appropriate sources to support medical AI research objectives. (2)
		Formulate well-defined research questions and hypotheses for medical AI research that are specific, testable, and aligned with clinical and methodological goals. (2)
		Apply scholarly rigor in analyzing AI research findings and producing evaluative academic reports. (1)
		Explain how physician knowledge and clinical experience contribute to the development of AI applications. (1)
		Interpret published research literature that employs machine learning methods. (1)
		Preprocess data for AI research by cleaning, transforming, and selecting relevant features. (1)
		Understand levels of evidence and trial endpoints in order to critically assess whether an AI intervention is justified. (1)
	Research Ecosystem and Impact (4)	Access, evaluate, use, share, and create information using information and communication technologies within the AI research ecosystem. (1)
		Analyze emerging trends and system-level impacts of artificial intelligence on healthcare organizations, including hospital operations and the medical AI market. (1)
		Describe novel methodologies for health data analysis using real-world data from diverse sources and explain their potential applications. (1)
		Provide domain-appropriate clinical consultation to support the development of medical artificial intelligence systems. (1)
	Applications to Translational Research (3)	Analyze applications of AI in biomedical research and public health, including personalized medicine, clinical trials, drug design, population monitoring, environmental risk assessment, epidemic surveillance, and disaster medicine. (1)
		Understand the utility of health data visualization and the various methods used to present health data effectively. (1)

Supplementary Table 4. Sensitivity Analysis – Rank-Order Stability

Domains are listed in rank order based on the number of unique studies contributing at least one eligible competency object, and competencies are ranked within their parent domain using the same study-support metric. Parenthetical annotations report study support and rank in the full dataset versus the sensitivity dataset (format: number of studies→ number of studies excluding editorial/opinion; frequency rank among all studies→frequency rank among sensitivity analysis; Δ, where Δ indicates change in rank with ↑ denoting improvement, ↓ denoting decline, and 0 denoting no change).

Domain	Competency
Theory & Foundations of AI (46→26; 1→1; Δ = 0)	AI Foundations and Core Concepts (20→13; 1→1; Δ = 0)
	Data Science and Health Data Fundamentals (14→9; 2→2; Δ = 0)
	Machine Learning Concepts and Paradigms (14→9; 2→2; Δ = 0)
	Statistical Foundations for AI in Medicine (9→7; 3→3; Δ = 0)
	Biases of AI Systems (8→4; 4→5; Δ = ↓1)
	Deep Learning and Neural Networks (7→5; 5→4; Δ = ↑1)
	Model Development, Training, and Optimization (7→5; 5→4; Δ = ↑1)
	Data Preprocessing and Feature Engineering (6→5; 6→4; Δ = ↑2)
	Natural Language Processing and Generative AI (6→4; 6→5; Δ = ↑1)
	Algorithms and Model Types (4→3; 7→6; Δ = ↑1)
	Evaluation, Error Analysis, and Explainability (3→3; 8→6; Δ = ↑2)
Clinical Applications of AI (34→24; 4→2; Δ = ↑2)	AI-Augmented Workflow Optimization (12→10; 1→1; Δ = 0)
	Clinical Use and Selection of AI Tools (12→9; 1→2; Δ = ↓1)
	AI-Based Clinical Decision Support (11→7; 2→3; Δ = ↓1)
	Diagnosis, Imaging, and Pathology (7→6; 3→4; Δ = ↓1)
	AI in Population Health and Value-Based Care (3→3; 4→5; Δ = ↓1)
AI Ethics (37→22; 2→3; Δ = ↓1)	Responsibility, Transparency, and Patient Rights (14→6; 1→1; Δ = 0)
	Bias and Equity (10→5; 2→2; Δ = 0)
	Allocation and Deployment of AI (5→1; 3→4; Δ = ↓1)
	Data Ethics (5→2; 3→3; Δ = 0)
Critical Appraisal of AI Output (35→19; 3→4; Δ = ↓1)	Opportunities and Limitations of AI systems (15→11; 1→1; Δ = 0)

	Analysis, Data Quality and Methodological Fit (14→7; 2→3; Δ = ↓1)
	Evidence Appraisal and Clinical Interpretation (12→8; 3→2; Δ = ↑1)
	Model Performance, Validation, and Error Characteristics (9→5; 4→4; Δ = 0)
	Implementation, Efficiency, and Quality Assurance (8→7; 5→3; Δ = ↑2)
AI Law & Regulation (26→14; 5→5; Δ = 0)	Data Privacy/Confidentiality and Protections (12→5; 1→2; Δ = ↓1)
	Regulatory Frameworks and Approval Processes (11→9; 2→1; Δ = ↑1)
	Data Stewardship (7→5; 3→2; Δ = ↑1)
	Legal Liability and Accountability (6→3; 4→3; Δ = ↑1)
	Consent and Socio-legal Obligations (5→2; 5→4; Δ = ↑1)
AI Professionalism in Healthcare (24→12; 6→6; Δ = 0)	Digital Communication, Literacy, and Conduct (15→8; 1→1; Δ = 0)
	Physician-Patient Communication and Shared Decision-making (11→4; 2→3; Δ = ↓1)
	Professional Identity, Roles, and Responsibilities (7→5; 3→2; Δ = ↑1)
	Reflective Use of AI in Patient-Centered Care (5→2; 4→4; Δ = 0)
Research & Innovation in AI (13→9; 7→7; Δ = 0)	Research Design and Methods (7→6; 1→1; Δ = 0)
	Research Ecosystem and Impact (4→3; 2→2; Δ = 0)
	Applications to Translational Research (3→1; 3→3; Δ = 0)

Supplementary Table 5. Domain Cross-Connectivity Matrix

Cells report the summed study-normalized cross-domain linkage weight from the row domain (source) to the column domain (target) (∑ = 37.00), derived from learning-objective–mediated competency linkages in the chord analysis. Larger values indicate stronger cross-domain coupling supported across included studies; values are directional (i.e., row→column may differ from column→row). Links are aggregated across all competency pairs spanning the corresponding domain pair; totals across all off-diagonal cells sum to the total cross-domain weight used to scale chord widths in **Figure 3**.

	AI Ethics	AI Law & Regulation	AI Professionalism in Healthcare	Clinical Applications of AI	Critical Appraisal of AI Output	Research & Innovation in AI	Theory & Foundations of AI
**AI Ethics**	0.00	1.07	0.43	0.86	1.05	0.00	0.86
**AI Law & Regulation**	1.20	0.00	0.94	0.01	0.20	0.13	0.13
**AI Professionalism in Healthcare**	2.75	0.45	0.00	0.45	0.72	0.36	2.47
**Clinical Applications of AI**	0.18	0.15	0.11	0.00	5.08	0.86	2.23
**Critical Appraisal of AI Output**	1.47	0.12	1.41	1.36	0.00	0.65	3.65
**Research & Innovation in AI**	0.09	0.00	0.30	0.00	0.60	0.00	1.11
**Theory & Foundations of AI**	0.05	0.15	1.05	0.20	1.42	0.69	0.00

Supplementary References. Included Sources of Evidence (*N* = 54)

1. Abid A, Murugan A, Banerjee I, Purkayastha S, Trivedi H, Gichoya J. AI Education for Fourth-Year Medical Students: Two-Year Experience of a Web-Based, Self-Guided Curriculum and Mixed Methods Study. JMIR Med Educ. 2024;10:e46500. doi:10.2196/46500
2. Alam F, Lim MA, Zulkipli IN. Integrating AI in medical education: embracing ethical usage and critical understanding. Front Med (Lausanne). 2023;10:1279707. doi:10.3389/fmed.2023.1279707
3. Alghamdi SA, Alashban Y. Medical science students' attitudes and perceptions of artificial intelligence in healthcare: A national study conducted in Saudi Arabia. Journal of Radiation Research and Applied Sciences. 2024;17(1):100815. doi:10.1016/j.jrras.2023.100815
4. Alkhaaldi SMI, Kassab CH, Dimassi Z, et al. Medical Student Experiences and Perceptions of ChatGPT and Artificial Intelligence: Cross-Sectional Study. JMIR Med Educ. 2023;9:e51302. doi:10.2196/51302
5. Al Shahrani A, Alhumaidan N, AlHindawi Z, et al. Readiness to Embrace Artificial Intelligence Among Medical Students in Saudi Arabia: A National Survey. Healthcare (Basel). 2024;12(24):2504. doi:10.3390/healthcare12242504
6. AlZaabi A, Masters K. Assessing medical students' readiness for artificial intelligence after pre-clinical training. BMC Med Educ. 2025;25(1):824. doi:10.1186/s12909-025-07008-x
7. Bansal M, Jindal A. Artificial intelligence in healthcare: Should it be included in the medical curriculum? A students' perspective. Natl Med J India. 2022;35(1):56-58. doi:10.25259/NMJI_208_20
8. Burwell JM. Preparing Tomorrow's Physicians: The Case for Machine Learning in Medical Education. J Med Syst. 2025;49(1):79. doi:10.1007/s10916-025-02214-y
9. Çalışkan SA, Demir K, Karaca O. Artificial intelligence in medical education curriculum: An e-Delphi study for competencies. PLoS One. 2022;17(7):e0271872. doi:10.1371/journal.pone.0271872
10. Chen J, Hou X, Lv Y, et al. What digital competencies should medical students in China possess in the AI era? Int J Med Inform. 2025;203:106043. doi:10.1016/j.ijmedinf.2025.106043

11. Cussat-Blanc S, Castets-Renard C, Monsarrat P. Doctors in Medical Data Sciences: A New Curriculum. Int J Environ Res Public Health. 2022;20(1):675. Published online January 2023. doi:10.3390/ijerph20010675
12. Henning PA, Henning J, Glück K. Artificial Intelligence: Its future in the health sector and its role for medical education. J Eur CME. 2021;10(1):2014099. doi:10.1080/21614083.2021.2014099
13. Hopson S, Mildon C, Hassard K, et al. Enhancing AI literacy in undergraduate pre-medical education through student associations: an educational intervention. BMC Med Educ. 2025;25(1):999. doi:10.1186/s12909-025-07556-2
14. Hu R, Fan KY, Pandey P, et al. Insights from teaching artificial intelligence to medical students in Canada. Commun Med (Lond). 2022;2(1):63. doi:10.1038/s43856-022-00125-4
15. Huang MY. Empowering medical students with AI literacy: A curriculum development journey. Med Educ. 2025;59(5):550-551. doi:10.1111/medu.15654
16. Imran N, Jawaid M. Artificial intelligence in medical education: Are we ready for it? Pak J Med Sci. 2020;36(5):857-859. doi:10.12669/pjms.36.5.3042
17. Jacobs SM, Lundy NN, Issenberg SB, Chandran L. Reimagining Core Entrustable Professional Activities for Undergraduate Medical Education in the Era of Artificial Intelligence. JMIR Med Educ. 2023;9:e50903. doi:10.2196/50903
18. Jamal A, Solaiman M, Alhasan K, Temsah MH, Sayed G. Integrating ChatGPT in Medical Education: Adapting Curricula to Cultivate Competent Physicians for the AI Era. Cureus. 2023;15(8):e43036. doi:10.7759/cureus.43036
19. Jong M. Transforming medical education in the AI era: Balancing technological expertise with humanistic care in tomorrow's doctors. Ann Acad Med Singap. 2024;53(12):754-757. doi:10.47102/annals-acadmedsg.2024242
20. Jotterand F, Bosco C. Artificial Intelligence in Medicine: A Sword of Damocles? J Med Syst. 2022;46(1):9. Published online December 11, 2021. doi:10.1007/s10916-021-01796-7
21. Katznelson G, Gerke S. The need for health AI ethics in medical school education. Adv Health Sci Educ Theory Pract. 2021;26(4):1447-1458. doi:10.1007/s10459-021-10040-3
22. Kimmerle J, Timm J, Festl-Wietek T, Cress U, Herrmann-Werner A. Medical Students' Attitudes Toward AI in Medicine and their Expectations for Medical Education. J Med Educ Curric Dev. 2023;10:23821205231219346. doi:10.1177/23821205231219346

23. Krive J, Isola M, Chang L, Patel T, Anderson M, Sreedhar R. Grounded in reality: artificial intelligence in medical education. JAMIA Open. 2023;6(2):ooad037. doi:10.1093/jamiaopen/ooad037
24. Laupichler MC, Tavakoli AA, Raupach T, Paech D. [Future skills-AI competencies for radiologists : Fostering AI knowledge and skills in undergraduate medical education]. Radiologie (Heidelb). 2024;64(4):316-320. doi:10.1007/s00117-023-01237-1
25. Lee YM, Kim S, Lee YH, et al. Defining Medical AI Competencies for Medical School Graduates: Outcomes of a Delphi Survey and Medical Student/Educator Questionnaire of South Korean Medical Schools. Acad Med. 2024;99(5):524-533. doi:10.1097/ACM.0000000000005618
26. Levingston H, Anderson MC, Roni MA. From Theory to Practice: Artificial Intelligence (AI) Literacy Course for First-Year Medical Students. Cureus. 2024;16(10):e70706. doi:10.7759/cureus.70706
27. Liaw W, Kueper JK, Lin S, Bazemore A, Kakadiaris I. Competencies for the Use of Artificial Intelligence in Primary Care. Ann Fam Med. 2022;20(6):559-563. doi:10.1370/afm.2887
28. Liu DS, Sawyer J, Luna A, et al. Perceptions of US Medical Students on Artificial Intelligence in Medicine: Mixed Methods Survey Study. JMIR Med Educ. 2022;8(4):e38325. doi:10.2196/38325
29. Lu H, Alhaskawi A, Dong Y, et al. Patient Autonomy in Medical Education: Navigating Ethical Challenges in the Age of Artificial Intelligence. Inquiry. 2024;61:469580241266364. doi:10.1177/00469580241266364
30. Masters K. Artificial Intelligence developments in medical education: a conceptual and practical framework. MedEdPublish (2016). 2020;9:239. doi:10.15694/mep.2020.000239.1
31. McCoy LG, Nagaraj S, Morgado F, Harish V, Das S, Celi LA. What do medical students actually need to know about artificial intelligence? NPJ Digit Med. 2020;3:86. doi:10.1038/s41746-020-0294-7
32. Meade SM, Salas-Vega S, Nagy MR, et al. A Pilot Remote Curriculum to Enhance Resident and Medical Student Understanding of Machine Learning in Healthcare. World Neurosurg. 2023;180:e142-e148. doi:10.1016/j.wneu.2023.09.012
33. Mihalas GI, Boru C, Cotoraci C. Teaching Artificial Intelligence to Medical Students. Stud Health Technol Inform. 2024;316:1505-1509. doi:10.3233/SHTI240700

34. Moldt JA, Festl-Wietek T, Fuhl W, et al. Assessing AI Awareness and Identifying Essential Competencies: Insights From Key Stakeholders in Integrating AI Into Medical Education. JMIR Med Educ. 2024;10:e58355. doi:10.2196/58355
35. Ng FYC, Thirunavukarasu AJ, Cheng H, et al. Artificial intelligence education: An evidence-based medicine approach for consumers, translators, and developers. Cell Rep Med. 2023;4(10):101230. doi:10.1016/j.xcrm.2023.101230
36. Oftring ZS, Deutsch K, Tolks D, Jungmann F, Kuhn S. Novel Blended Learning on Artificial Intelligence for Medical Students: Qualitative Interview Study. JMIR Med Educ. 2025;11:e65220. doi:10.2196/65220
37. Onoue T, Asada Y, Imafuku R, et al. Developing competencies relating to information science and technology in Japanese undergraduate medical education. Med Teach. 2024;46(sup1):S31-S37. doi:10.1080/0142159X.2024.2385199
38. Paranjape K, Schinkel M, Nannan Panday R, Car J, Nanayakkara P. Introducing Artificial Intelligence Training in Medical Education. JMIR Med Educ. 2019;5(2):e16048. doi:10.2196/16048
39. Park SH, Pinto-Powell R, Thesen T, et al. Preparing healthcare leaders of the digital age with an integrative artificial intelligence curriculum: a pilot study. Med Educ Online. 2024;29(1):2315684. doi:10.1080/10872981.2024.2315684
40. Saroha S. Artificial Intelligence in Medical Education: Promise, Pitfalls, and Practical Pathways. Adv Med Educ Pract. 2025;16:1039-1046. doi:10.2147/AMEP.S523255
41. Schubert T, Oosterlinck T, Stevens RD, Maxwell PH, van der Schaar M. AI education for clinicians. EClinicalMedicine. 2025;79:102968. doi:10.1016/j.eclinm.2024.102968
42. Seth P, Hueppchen N, Miller SD, et al. Data Science as a Core Competency in Undergraduate Medical Education in the Age of Artificial Intelligence in Health Care. JMIR Med Educ. 2023;9:e46344. doi:10.2196/46344
43. Shimizu I, Kasai H, Shikino K, et al. Developing Medical Education Curriculum Reform Strategies to Address the Impact of Generative AI: Qualitative Study. JMIR Med Educ. 2023;9:e53466. doi:10.2196/53466
44. Singla R, Pupic N, Ghaffarizadeh SA, et al. Developing a Canadian artificial intelligence medical curriculum using a Delphi study. NPJ Digit Med. 2024;7(1):323. doi:10.1038/s41746-024-01307-1
45. Sumner B, Martin R, Gladman T, Wilkinson TJ, Grainger R. Understanding the gap: a balanced multi-perspective approach to defining essential digital health competencies for medical graduates. BMC Med Educ. 2025;25(1):682. doi:10.1186/s12909-025-07194-8

46. Theros J, Soetikno A, Liebovitz D. Foundations of AI for future physicians: A practical, accessible curriculum. Med Teach. 2025;47(10):1601-1603. doi:10.1080/0142159X.2025.2463492
47. Thomae AV, Witt CM, Barth J. Integration of ChatGPT Into a Course for Medical Students: Explorative Study on Teaching Scenarios, Students' Perception, and Applications. JMIR Med Educ. 2024;10:e50545. doi:10.2196/50545
48. Tong W, Zhang X, Zeng H, Pan J, Gong C, Zhang H. Reforming China's Secondary Vocational Medical Education: Adapting to the Challenges and Opportunities of the AI Era. JMIR Med Educ. 2024;10:e48594. doi:10.2196/48594
49. Triola MM, Rodman A. Integrating Generative Artificial Intelligence Into Medical Education: Curriculum, Policy, and Governance Strategies. Acad Med. 2025;100(4):413-418. doi:10.1097/ACM.0000000000005963
50. Waibel AM, Bischoff M. [Digital competencies in medical studies: results of an interdisciplinary course]. HNO. 2024;72(3):161-165. doi:10.1007/s00106-023-01411-w
51. Waldock WJ, Lam G, Baptista A, Walls R, Sam AH. Which curriculum components do medical students find most helpful for evaluating AI outputs? BMC Med Educ. 2025;25(1):195. doi:10.1186/s12909-025-06735-5
52. Weidener L, Fischer M. Artificial Intelligence Teaching as Part of Medical Education: Qualitative Analysis of Expert Interviews. JMIR Med Educ. 2023;9:e46428. doi:10.2196/46428
53. Weidener L, Fischer M. Proposing a Principle-Based Approach for Teaching AI Ethics in Medical Education. JMIR Med Educ. 2024;10:e55368. doi:10.2196/55368
54. Zhong JY, Fischer NL. Commentary: The desire of medical students to integrate artificial intelligence into medical education: An opinion article. Front Digit Health. 2023;5:1151390. doi:10.3389/fdgth.2023.1151390
